# Fibrocytes boost tumor-supportive phenotypic switches in the lung cancer niche via the endothelin system

**DOI:** 10.1038/s41467-022-33458-8

**Published:** 2022-10-14

**Authors:** Andreas Weigert, Xiang Zheng, Alina Nenzel, Kati Turkowski, Stefan Günther, Elisabeth Strack, Evelyn Sirait-Fischer, Eiman Elwakeel, Ivan M. Kur, Vandana S. Nikam, Chanil Valasarajan, Hauke Winter, Alexander Wissgott, Robert Voswinkel, Friedrich Grimminger, Bernhard Brüne, Werner Seeger, Soni Savai Pullamsetti, Rajkumar Savai

**Affiliations:** 1 Goethe-University Frankfurt, Faculty of Medicine, Institute of Biochemistry I, Frankfurt am Main, Germany; 2 Frankfurt Cancer Institute (FCI), Goethe University, and German Cancer Consortium (DKTK), Partner Site Frankfurt, Frankfurt am Main, Germany; 3 Max Planck Institute for Heart and Lung Research, Member of the German Center for Lung Research (DZL), Member of the Cardio-Pulmonary Institute (CPI), Bad Nauheim, Germany; 4 Translational Lung Research Center (TLRC), Member of the DZL; Department of Thoracic Surgery, Thorax klinik at the University Hospital Heidelberg, Heidelberg, Germany; 5 Department of Internal Medicine, Justus-Liebig University Giessen, Member of the DZL, Member of CPI, Giessen, Germany; 6 Institute for Lung Health (ILH), Justus Liebig University, Giessen, Germany

**Keywords:** Cancer microenvironment, Cellular imaging, Non-small-cell lung cancer

## Abstract

Fibrocytes are bone marrow–derived monocytic cells implicated in wound healing. Here, we identify their role in lung cancer progression/ metastasis. Selective manipulation of fibrocytes in mouse lung tumor models documents the central role of fibrocytes in boosting niche features and enhancing metastasis. Importantly, lung cancer patients show increased number of circulating fibrocytes and marked fibrocyte accumulation in the cancer niche. Using double and triple co-culture systems with human lung cancer cells, fibrocytes, macrophages and endothelial cells, we substantiate the central features of cancer-supporting niche: enhanced cancer cell proliferation and migration, macrophage activation, augmented endothelial cell sprouting and fibrocyte maturation. Upregulation of endothelin and its receptors are noted, and dual endothelin receptor blockade suppresses all cancer-supportive phenotypic alterations via acting on fibrocyte interaction with the cancer niche. We thus provide evidence for a crucial role of fibrocytes in lung cancer progression and metastasis, suggesting targets for treatment strategies.

## Abstract (web summary)

Fibrocytes are monocyte-derived cells implicated in wound healing. Here, the authors utilise single cell RNA-seq, genetic ablation and multiplexed imaging to identify a fibrocyte population in lung cancer models, and use human lung cancer coculture systems to highlight their potential to modulate microenvironmental niche and sensitivity to endothelin blockade.

## Introduction

Fibrocytes are monocyte-derived cells that have features of both macrophages and fibroblasts. They are unique because they co-express hematopoietic and progenitor cell markers (CD45 and CD34, respectively) and produce extracellular matrix proteins (Collagen1A1, Vimentin)^1,2^. Despite their low abundance (they constitute < 1% of the adult circulating leukocyte pool), they strongly promote wound healing by migrating to injured areas where they differentiate into activated fibroblasts, such as contractile myofibroblasts^3^ that appear in many fibrotic lesions including those of the lung^4^. In addition, increased numbers of fibrocytes are found in human lung pathological states that are characterized by chronic immune cell-driven inflammation such as asthma, pulmonary fibrosis^5–7^ and pulmonary hypertension^8–10^. The presence and role of circulating fibrocytes^11,12^ in lung cancer are, however, unknown.

Recent studies indicate that fibrocytes have a role extending far beyond tissue remodeling and extracellular matrix production. For example, fibrocytes contribute to innate immunity by expressing antimicrobial factors^13^ and to adaptive immunity by antigen presentation^14,15^. In addition, circulating fibrocytes can prompt an influx of Ly-6C^+^ monocytes into the lung^16^ and can exert pro-inflammatory effects via their macrophage-like properties^17^. These findings suggest that fibrocytes might also be involved in cancer promotion. In contrast, fibrocytes that are positive for fibroblast activation protein-alpha are implicated as mediators of tumor immune escape^18^.

Our recent work suggests that systemic depletion of monocytes/macrophages leads to decreased tumor size, tumor cell proliferation and migration in various mouse models of lung cancer^19^. Broadening the scope to fibrocytes, we examined the functional contribution of these cells to lung cancer progression and metastasis by first more deeply characterizing fibrocytes via single cell transcriptomics, followed by selective in vivo−depletion techniques as well as co-application of fibrocytes with lung cancer cells in both xenograft and KRas-driven lung tumorigenesis models. In addition, we assessed the clinical significance of circulating and tumor-microenvironmental fibrocytes in lung cancer patients. Double and triple co-culture systems were used to dissect the impact of fibrocytes on cancer cell biology, macrophage phenotype and angiogenesis. Further, based on our observed upregulation of the endothelin system in the lung cancer niche, we probed the impact of endothelin receptor antagonism on these features.

## Results

### Single-cell RNA-seq identifies a combination of fibrocyte specific marker signature

**Fig. 1:**
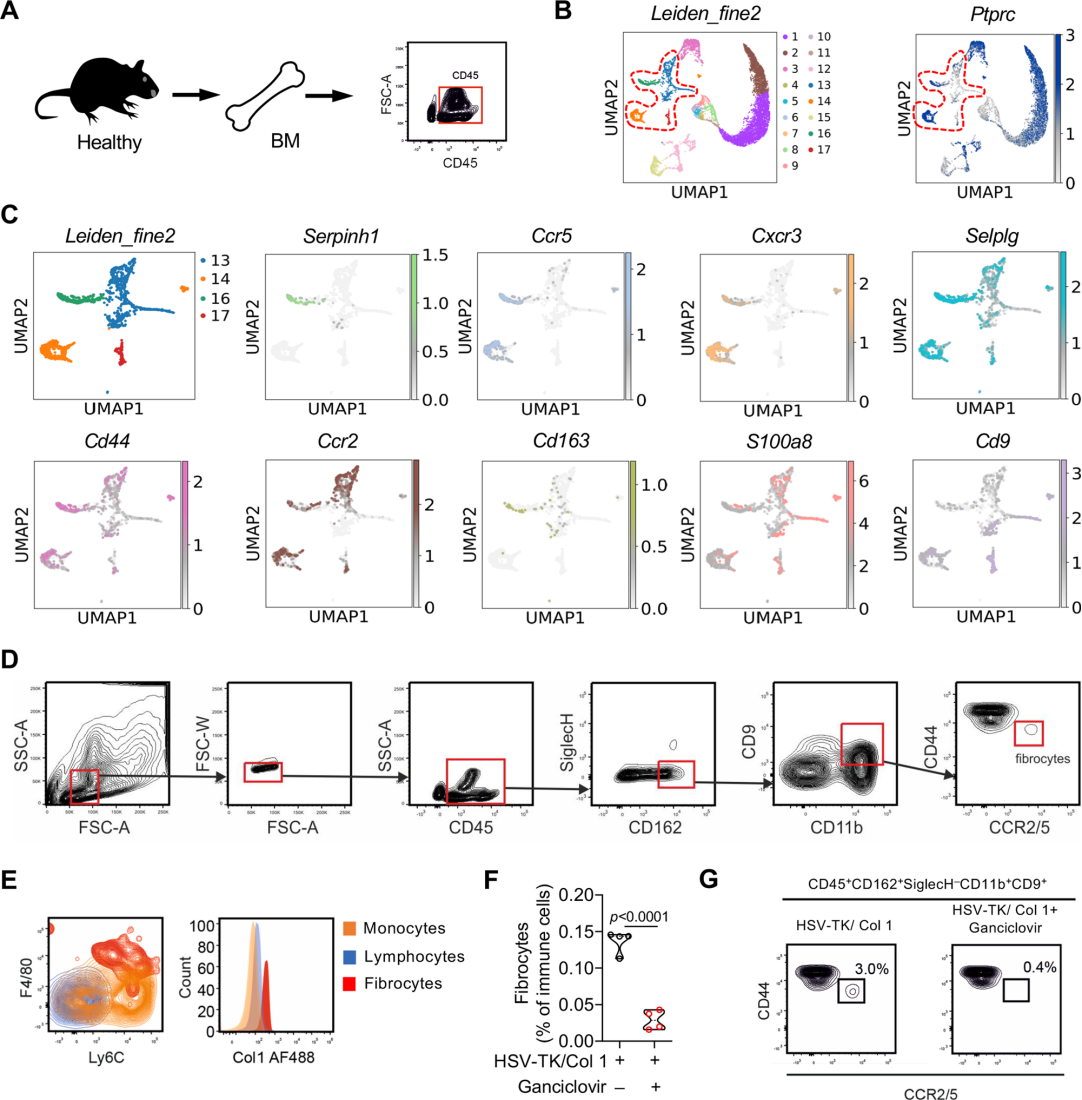
Single-cell RNA-seq identifies a combination of fibrocyte specific markers. **A** Schematic diagram of CD45^+^ cell isolation from the bone marrow of a C57BL/6 mouse. **B** Uniform manifold approximation and projection (UMAP) representation of the data clustered by the Leiden algorithm revealing 17 clusters (left). Expression of *Prprc* (CD45) is shown in the panel on the right. **C** UMAP representation of all cells belonging to cluster 13, 14, 16, and 17 by the Leiden algorithm. Expression of *Serpinh1, Ccr5, Cxcr3, Selplg, Cd44, Ccr2, Cd163, S100a8* and *Cd9* are shown. **D** Representative FACS contour plots of fibrocytes (CD45^+^SiglecH^–^, CD162^+^CD9^+^CD11b^+^CD44^+^CCR2/5^+^) in bone marrow of C57BL/6 mice. **(E)** Representative FACS contour plot and histogram showing Ly6C, F4/80, and Col1 expression in monocytes (CD11b^+^/Ly6G^-^; orange), lymphocytes (CD11b^–^; blue) and fibrocytes (CD45^+^/SiglecH^–^, CD162^+^CD9^+^CD11b^+^CD44^+^CCR2/5^+^; red). **F** Quantification of fibrocytes in fibrocyte-depleted mice (HSV-TK/Col1+Ganciclovir) and control mice (HSV-TK/Col1), *n* = 4. *p*-values were determined by two-tailed unpaired t–test with Welch’s correction. **G** Representative FACS contour plots of fibrocytes in fibrocyte-depleted (HSV-TK/Col1+Ganciclovir) and control (HSV-TK/Col1) mice. Source data are provided in the source data file.

Fibrocytes are defined as monocytic cells expressing collagen-1 (Col1). To characterize these cells more deeply, we FACS sorted CD45^+^ cells (Fig. 1A) from C57BL/6 mice bone marrow and performed single cell RNA sequencing (scRNA-seq). After quality control and accounting for technical noise, single-cell transcriptomic profiles for 10937 cells and 19602 genes were considered for the analysis. A Uniform Manifold Approximation and Projection (UMAP) algorithm showed 17 cell populations present in bone marrow and all cell populations expressed P*tprc* (CD45) (Fig. 1B). These 17 cell clusters included lymphocytes (cluster 12, 14, 15), mast cells (cluster 17), monocytes (cluster 3, 13), granulocytes (clusters 1, 2), and their progenitors (clusters 4-10) (Supplementary Data 1). Collagens were not or only poorly expressed in BM cells, due to the limited depth of scRNA-seq (Supplementary Fig. 1A). To identify fibrocyte-containing clusters, we therefore analyzed the expression of Serpinh1 (Hsp47), an ER-resident chaperone that is essential for the proper assembly of the triple-helical procollagen molecules, whose expression levels correlate strictly with the amounts of collagen being synthesized^20^. *Serpinh1* expression was mainly observed in cluster 16, which also contained plasmacytoid dendritic cells (pDCs) (Fig. 1C, Supplementary Data 1). Further screening for the expression of genes associated with human fibrocytes (*Cd44, Cd163, and S100a8*)^21^ ascertained the expression of fibrocyte-associated genes in cluster 16 (Fig. 1C and Supplementary Fig. 1B). Besides known fibrocyte markers, cluster 16 was also characterized by the expression of *Ccr2, Ccr5, Cxcr3, Selplg* and *Cd9* (Fig. 1C and Supplementary Fig. 1B). Molecules that regulate glycosylation of hydroxylated lysines in collagen (Collagen Beta (1-O) Galactosyltransferase 1 (*Colgalt1*)) and collagen cross-link via lysyl hydroxylation (procollagen-lysine, 2-oxoglutarate 5-dioxygenase (*Plod1*−3)) were also expressed in cluster 16, but *Serpinh1* was the main molecule that differentiates between cluster 14 and 16. Moreover, cells in cluster 14 did not express *Cd68*, a marker of the myeloid lineage in mice, excluding them as potential fibrocytes. Thus, we considered cluster 16 as fibrocytes (Supplementary Fig. 1B). To validate if this strategy allowed fibrocyte identification, we developed a flow cytometry panel containing these markers and performed FACS analysis of C57BL/6 bone marrow with or without depleting Col1-expressing cells. Expression of CD45, CD162 (Selplg), CD9, CD11b, CD44, CCR2 and CCR5 was found in a small, discrete populations of cells co-expressing the monocyte/macrophage markers Ly6C and F4/80, but not expressing the plasmacytoid DC marker SiglecH. Importantly, these cells selectively expressed Col1 compared to other monocytic cells and lymphocytes (Fig. 1D, E), corresponding to the definition of fibrocytes. Transgenic mice expressing the herpes simplex virus thymidine kinase (HSV-TK) suicidal gene under the control of the Col1 promoter (HSV-TK/Col1 C57BL/6 mice) allowed depletion of Col1^+^ cells with ganciclovir^22^ (Supplementary Fig. 1C). Notably, only fibrocytes were significantly decreased in the bone marrow of ganciclovir treated HSV-TK/Col1 mice compared to non-treated HSV-TK/Col1 mice (Fig. 1F, G), while other cell populations in the bone marrow were not affected (Supplementary Fig. 1D). Thus, we were able to selectively deplete bone marrow-derived Col1 producing cells corresponding to fibrocytes to test their involvement in lung cancer.

**Fig. 2:**
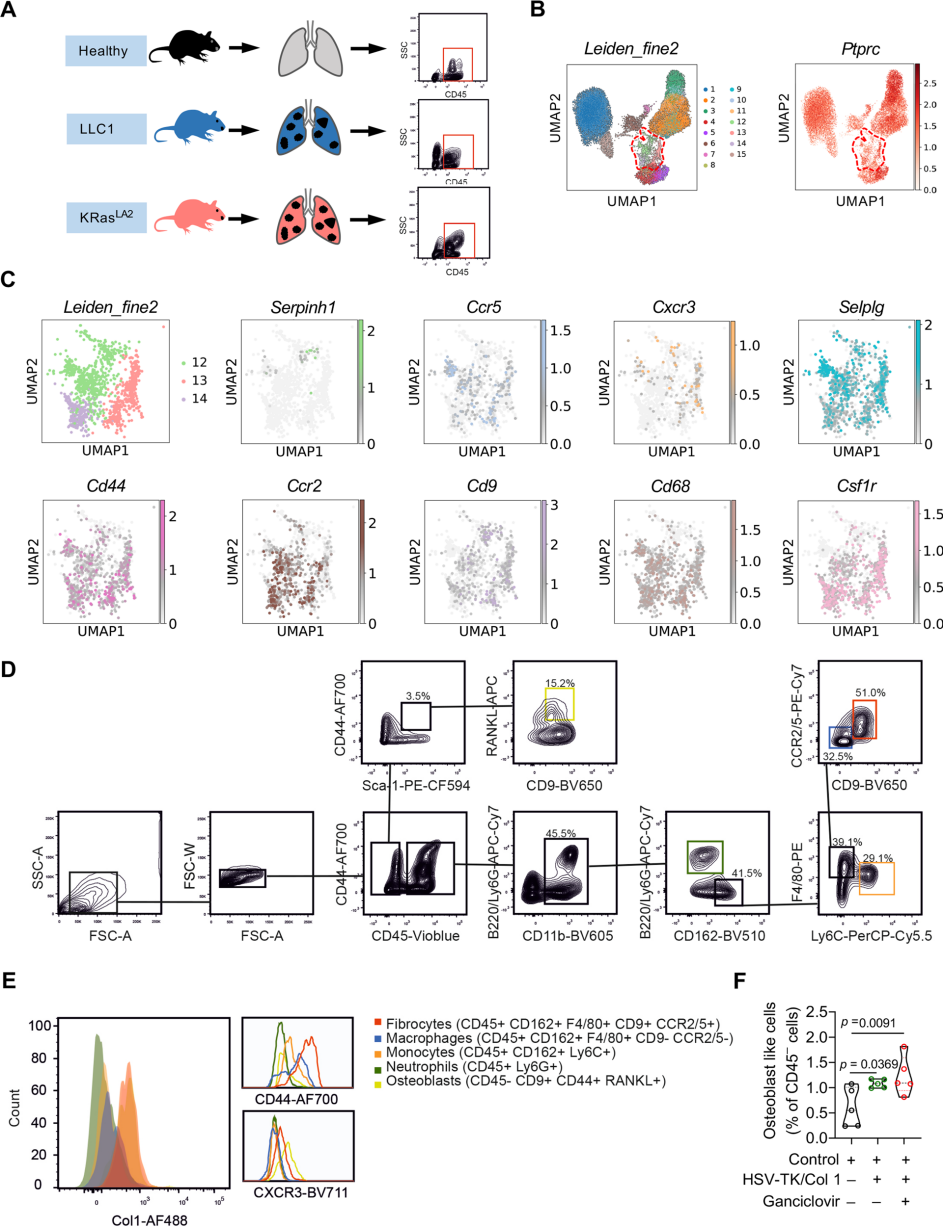
Lung cancer CD45+ cells scRNA-seq validates identified fibrocyte specific markers. **A** Schematic diagram of CD45^+^ cell isolation from the healthy, LLC1 and KRas^LA2^ lung tumor models. **B** UMAP representation of the data clustered by the Leiden algorithm revealing 15 clusters (left). Expression of *Prprc* (CD45) is shown in the panel on the right. **C** UMAP representation of all cells belonging to cluster 12, 13, and 14 by the Leiden algorithm. Expression of *Serpinh1, Ccr5, Cxcr3, Selplg, Cd44, Ccr2, Cd9, Cd68, and Csf1r* are shown. **D** Gating strategy used to identify fibrocytes, monocytes, macrophages, neutrophils and osteoblast-like cells. Representative flow-cytometric analysis used to identify fibrocytes (CD45^+^CD11b^+^CD162^+^F4/80^+^CCR2/5^+^CD9^+^CD44^+^Col1^+^), monocytes (CD45^+^CD11b^+^/Ly6C^+^), macrophages (CD45^+^CD11b^+^CD162^+^F4/80^+^CCR2/5lo/^–^CD9^–^), neutrophils (CD45^+^CD11b^+^Ly6G^+^) and osteoblast-like cells (CD45^–^CD44^+^CD9^+^ RANKL^+^). **E** Representative FACS histograms show expression of Col-1, CD44, CXCR3 in fibrocytes (CD45^+^CD162^+^F4/80^+^CD9^+^CCR2/5^+^), macrophages (CD45^+^CD162^+^F4/80^+^CD9^–^CCR2/5^–^), monocytes (CD45^+^CD162^+^Ly6C^+^), neutrophils (CD45^+^Ly6G^+^) and osteoblast-like cells (CD45^–^CD9^+^CD44^+^RANKL^+^). **F** Quantification of osteoblast-like cells (CD45^–^CD9^+^CD44^+^RANKL^+^) in the i.v. lung tumor model from fibrocyte-depleted (HSV-TK/Col1+Ganciclovir), HSV-TK/Col1 compared with control lung tumor tissue by FACS analysis. *n* = 5. *p*-values were determined by One-way ANOVA with Fisher’s LSD test. Source data are provided in the source data file.

To identify fibrocyte-containing clusters in lung tumors, we performed scRNA-seq of CD45^+^ cells from LLC1 and KRas^LA2^ lung tumors. This analysis revealed 15 cell populations present in lung tumors and all cell populations expressed *Ptprc* (Fig. 2A, B). Further we analyzed the expression of Serpinh1 and genes associated with fibrocytes (Cd44, Ccr2, Ccr5, Cxcr3, Selplg and Cd9) (Fig. 2C). As shown in Fig. 2C, cluster 12 expressed most of these markers including macrophage markers (Cd68, Csf1r). FACS analysis of lung tumors with flow cytometry panel containing these markers confirmed the presence of fibrocytes. Fibrocytes that were identified in lung tumors as CD45^+^CD44^+^CD162^+^CD11b^+^F4/80^+^CCR2/5^+^CD9^+^ cells had lost the monocyte marker Ly6C compared to bone marrow fibrocytes, indicating a maturation process in the cancer microenvironment, reminiscent of monocyte to macrophage differentiation in tumors (Fig. 2D, E). Lung tumor fibrocytes expressed Col1 at similar levels as stromal CD45^–^ osteoblast-like cells, while other myeloid cells did not (Fig. 2E). Importantly, although osteoblast lineage cells can be transferred from the host during bone marrow transfer and CD45^–^ fibrocyte-like cells were previously described to contribute to wound healing^23^, ganciclovir did not deplete these CD45^–^cells in lung tumors (Fig. 2F).

### Fibrocyte depletion results in reduced primary tumor growth and metastasis

**Fig. 3:**
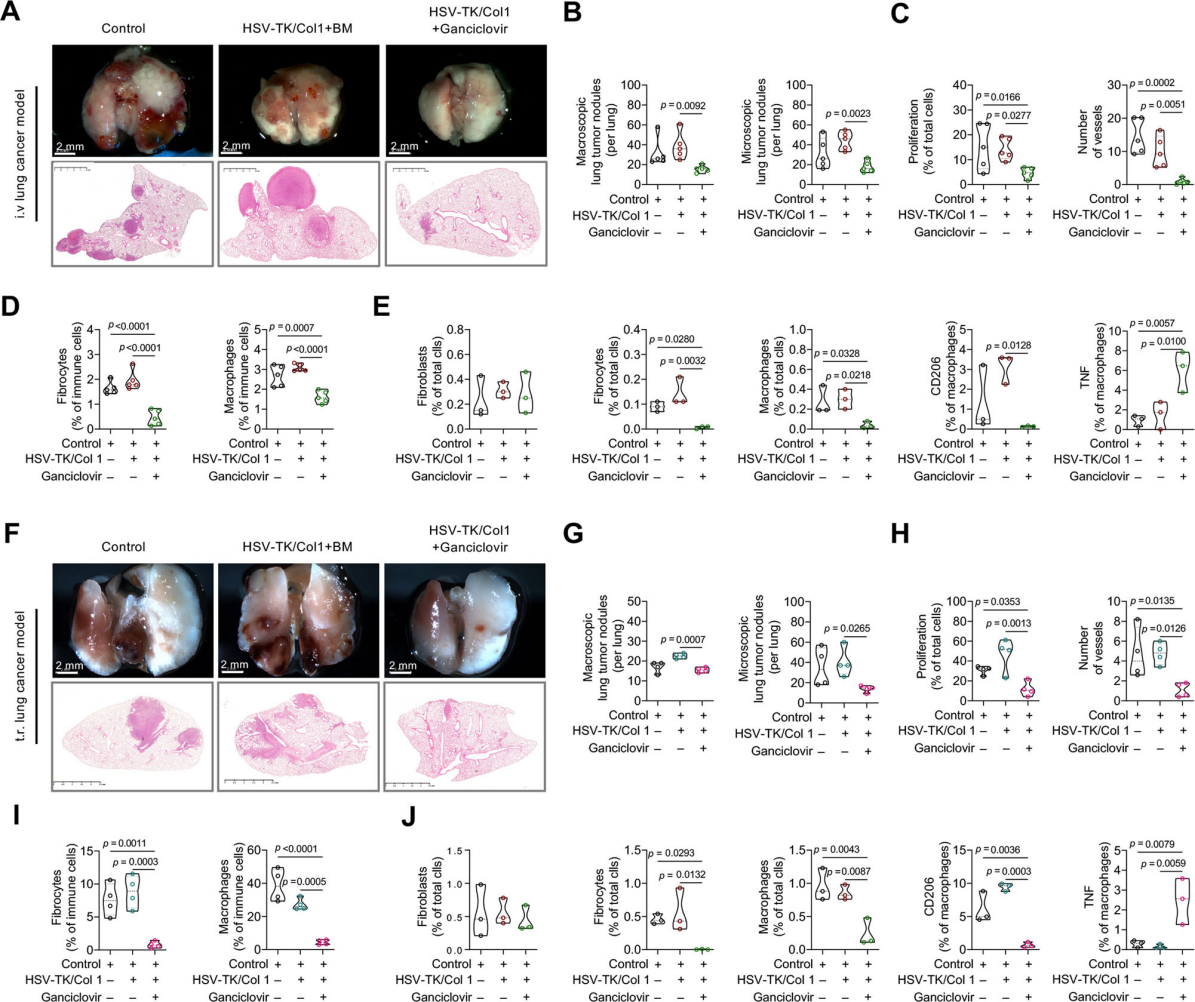
Fibrocyte depletion reduces lung tumor progression in LLC1 syngeneic mouse lung tumor models. **A** Photographs (upper panel) of whole lung (scale bar, 2 mm) and Hematoxylin and eosin (H&E) stainings (down panel) of lung sections (scale bar, 2.5 mm) that represents intravenous (i.v.) lung metastasis model in fibrocyte-depleted (HSV-TK/Col1+Ganciclovir) and control mice (HSV-TK/Col1+BM and control), *n* = 5. **B** Quantification of macroscopic and microscopic lung tumor nodules from fibrocyte-depleted and control lung tumors, *n* = 5. **C** Quantification of PCNA^+^ proliferating cells (*n* = 5, 7 images per lung tumor) and vWF^+^ vessels (*n* = 5, 5 images per lung tumor) from fibrocyte-depleted and control lung tumors by immunohistochemistry. **D** Quantification of fibrocytes (CD45^+^CD162^+^F4/80^+^CD9^+^CCR2/5^+^) and macrophages (CD45^+^CD162^+^F4/80^+^CD9^–^CCR2/5^–^) from fibrocyte-depleted and control lung tumors by FACS analysis, *n* = 5. **E** Quantification of fibroblasts (CD45^–^CD163^–^Col1^+^), fibrocytes (CD45^+^CD163^+^CCR2^+^Col1^+^), and macrophages (CD45^+^CD163^+^CCR2^–^Col1^–^), M2-like (CD206^+^) and M1-like (TNF^+^) macrophages from fibrocyte-depleted and control lung tumors by multiplex immunofluorescence, *n* = 3. **F** Representative photographs (upper panel) of whole lung (scale bar, 2 mm) and H&E stainings (down panel) of lung sections (scale bar, 2.5 mm) to represent tumor relapse (t.r.) lung metastasis model in fibrocyte-depleted and control mice, *n* = 5. **G** Quantification of macroscopic and microscopic lung tumor nodules from fibrocyte-depleted and control lung tumors, *n* = 5. **H** Quantification of PCNA^+^ proliferating cells (*n* = 4, 7 images per lung tumor) and vWF^+^ vessels (*n* = 4, 5 images per lung tumor) from fibrocyte-depleted and control lung tumors by immunohistochemistry. **I** Quantification of fibrocytes (CD45+CD162+F4/80+CD9+CCR2/5+) and macrophages (CD45^+^CD162^+^F4/80^+^CD9^–^CCR2/5^–^) with the markers described above from fibrocyte-depleted and control lung tumors by FACS analysis, *n* = 4. **J** Quantification of fibroblasts (CD45^-^CD163^–^Col1^+^), fibrocytes (CD45^+^CD163^+^CCR2^+^Col1^+^), macrophages (CD45^+^CD163^+^CCR2^–^Col1^–^), M2-like (CD206^+^) and M1-like (TNF^+^) macrophages from fibrocyte-depleted and control lung tumors by multiplex immunofluorescence, *n* = 3. **B**–**E, G**–**J** *p*-values were determined using One-way ANOVA with Fisher’s LSD test. Source data are provided in the source data file.

To determine the functional contribution of fibrocytes in lung cancer, bone marrow chimeras were generated, whereby bone marrow from HSV-TK/Col1 C57BL/6 mice was transplanted into lethally irradiated wild-type C57BL/6 mice (Supplementary Fig. 2A). Therefore, toxic effects of ganciclovir on host cells were excluded, and depletion was restricted to bone marrow−derived Col1^+^ cells (i.e., fibrocytes). We used this approach in three different mouse lung cancer models based on the Lewis lung carcinoma line 1 (LLC1) cell line^19,24^. Notably, fibrocyte depletion in subcutaneous (s.c.) LLC1 tumor−bearing mice led to a significant reduction in tumor size and weight (Supplementary Fig. 2B, C). LLC1 tumor−bearing mice with fibrocyte depletion showed reduced cell proliferation, angiogenesis and a smaller number of macrophages in their tumor tissue as assessed by histology (Supplementary Fig. 2D–F). Furthermore, fibrocyte-depletion in both intravenous (i.v.) and tumor relapse (t.r.) LLC1 lung tumor mice^19,25^ resulted in a reduced number of macroscopic and microscopic metastatic tumor nodules (Fig. 3A, B, F, G). Similar to primary s.c. tumors, both i.v. and t.r. metastatic tumors (macroscopic and microscopic) showed an association between fibrocyte depletion and reduced number of proliferating cells and vessels (Fig. 3C, H). In addition, fibrocyte depletion not only resulted in reduced numbers of fibrocytes, but also reduced the number of macrophages and their polarization towards an M2-like differentiation phenotype (CD206) without influencing fibroblasts (Fig. 3D, E, I, J and Supplementary Fig. 2G, H).

### Fibrocyte depletion suppresses KRas-driven lung tumorigenesis

KRas^LA2^ mice develop spontaneous lung carcinoma with full penetrance that reliably recapitulates the clinical and histopathological features of the human disease^26^. These mice develop adenomatous hyperplasia 1 week after birth and a considerable tumor load over a 5-month period^26^. To investigate the role of fibrocytes in lung tumor development, we crossed the HSV-TK/Col1 mice with KRas^LA2^ mice. Daily ganciclovir injections were initiated when mice developed microscopic nodules (3 months), and further progression to lung adenocarcinomas was quantified. However, bone marrow transfer was not performed in these animals due to anticipated off-target effects on transgenic tumor growth during irradiation^27,28^ Compared with control groups (KRas^LA2^ and KRas^LA2^ + ganciclovir mice), ganciclovir-treated HSV-TK/Col1 + KRas^LA2^ mice showed significantly fewer lung tumors at 5 months of age, as assessed either by visual inspection or by histological analysis (Supplementary Fig. 3A). Correspondingly, reduced numbers of both macroscopic and microscopic lung tumor nodules were measured in these mice (Supplementary Fig. 3B). In agreement with a reduced tumor burden, ganciclovir-treated HSV-TK/Col1 + KRas^LA2^ mice showed significantly decreased numbers of proliferating cells, blood vessels (Supplementary Fig. 3C), fibroblasts, fibrocytes and macrophages in the tumor niches, accompanied by reduced M2-like macrophage and increased M1-like macrophage markers (Supplementary Fig. 3D, E), all compared with KRas^LA2^ and KRas^LA2^ + ganciclovir control mice.

### Fibrocytes are increased in lung and peripheral blood of patients with lung cancer

**Fig. 4:**
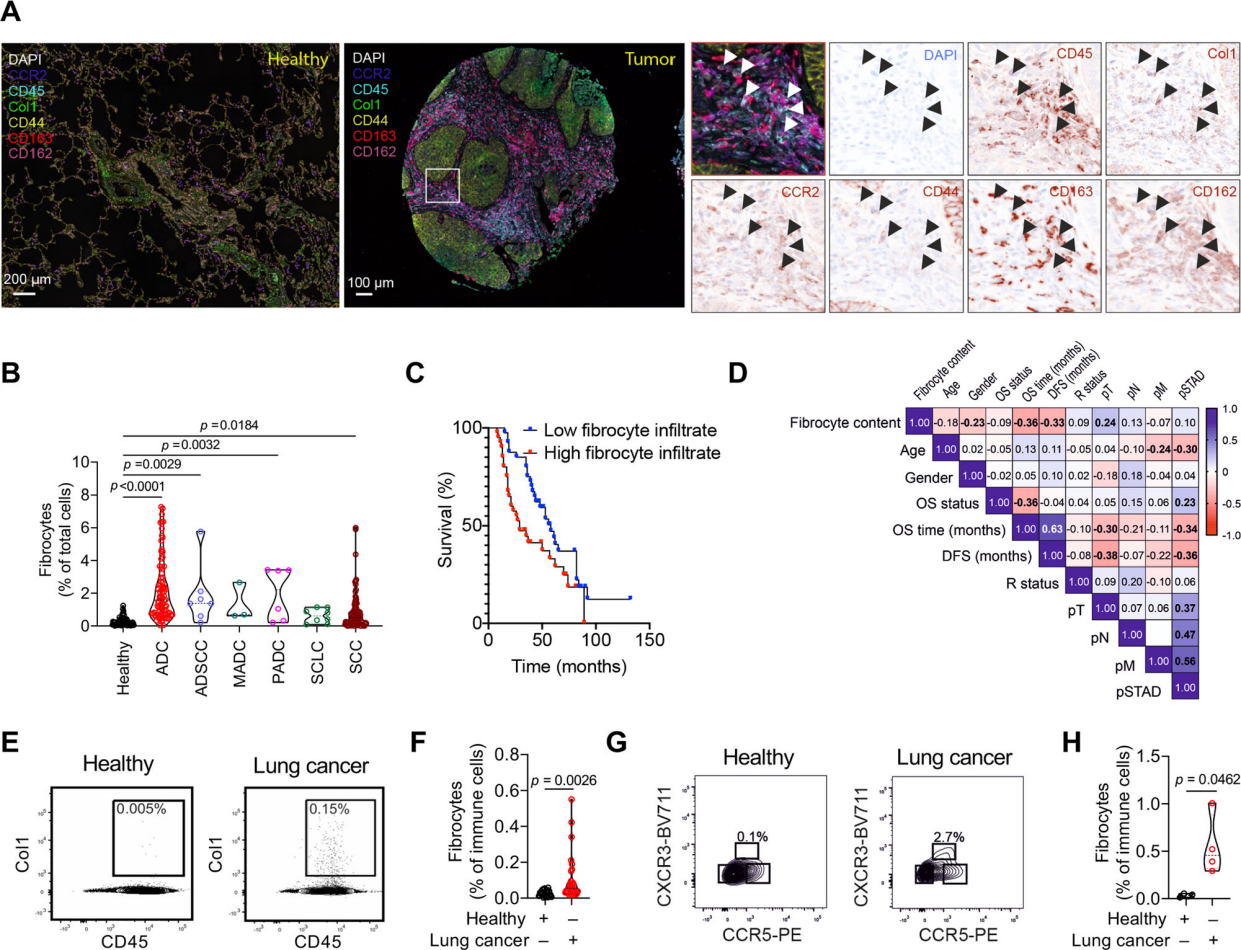
High fibrocyte infiltrates negatively correlate with overall and disease-free survival time and positively correlate with pT. **A**, **B** Representative multispectral images of tissue samples from healthy lung and lung tumor tissue (left panel). Tissues were stained with CD45 (cyan) and Col1 (green), CD44 (yellow) and CD163 (red), CCR2 (blue) and CD162 (magenta) antibodies. Nuclei were counterstained with DAPI (white). Right side panel represents zoomed-in images from the squared area. Arrows indicate fibrocytes (CD45^+^Col1^+^CD44^+^CD163^+^CCR2^+^CD162^+^) scale bar, 200 µm (healthy), 100 µm (tumor). Quantification of fibrocytes in lung tissue from healthy donors (*n* = 59) relative to individuals with adenocarcinoma (ADC, *n* = 86), adenosquamous carcinoma (ADSCC, *n* = 7), mucous adenocarcinoma (MADC, *n* = 3), Papillary adenocarcinoma (PADC, *n* = 6), small cell carcinoma (SCLC, *n* = 8) and squamous cell carcinoma (SCC, *n* = 94) using a TMA. *P*-values were determined using One-way ANOVA with Fisher’s LSD test. **C** Kaplan-Meier plots reveal patient survival according to fibrocyte infiltrates in the lung tumor tissue (*n* = 80). **D** Heatmap showing the correlation of tissue categories or cell subsets with clinical parameters. Non-parametric correlation analysis (Spearman) was performed, and *p*-values for survival analyses were calculated using log-rank test (*n* = 80). Fields with significant correlations (*p* < 0.05) are indicated in bold script. **E** Representative contour plots for identification of circulating CD45^+^Col1^+^ fibrocytes from PBMCs of healthy donors and lung cancer patients. **F** Relative quantification of CD45^+^Col1^+^ fibrocytes from PBMCs of healthy donors (*n* = 20) and lung cancer patients (*n* = 30). *p*-values were determined using two-tailed unpaired *t*-test with Welsh’s correction. **G** Representative contour plots for identification of circulating (CD45^+^CD33^+^CD162^+^CD44^+^CCR2^/^5^+^CXCR3^+^Col1^+^) fibrocytes from PBMCs of healthy donors and lung cancer patients. **H** Relative quantification of fibrocytes from PBMCs of healthy donors (*n* = 4) and lung cancer patients (*n* = 4). *p*-values were determined using two-tailed unpaired *t*-test. Source data are provided in the source data file.

To quantify fibrocytes in the lungs and the circulating blood of patients with lung cancer, we analyzed 284 lung cancer tissues using Phenoptics multicolor immunofluorescence with a condensed marker panel and PBMCs using FACS analysis. Importantly, increased accumulation of fibrocytes (CD45^+^Col1^+^CCR2^+^CD44^+^CD163^+^CD162^+^) was observed in tumor tissue of all lung cancer types, including non-small cell lung carcinoma (adenocarcinoma (ADC), adenosquamous cell carcinoma (ADSCC), mucous adenocarcinoma (MADC), papillary adenocarcinoma (PADC), and squamous cell carcinoma (SCC), as compared with healthy lung tissues (Fig. 4A, B). Though fibrocyte numbers were unchanged in MADC and SCLC tumor types, a limited number of patient samples were analyzed in some of these tumor types (Fig. 4B). Importantly, the number of fibrocytes correlated with survival (i.e., overall free survival (OS) and disease-free survival (DFS)) and positively correlated with tumor status (pT; Fig. 4C, D). Additionally, FACS analysis of CD45^+^Col1^+^ cells (Fig. 4E, F) showed significantly increased numbers of circulating fibrocytes in PBMCs isolated from patients with lung cancer compared with healthy control samples. These data were further confirmed using a more elaborate marker panel based on mouse fibrocyte markers (CD45^+^CD44^+^CD162CD33^+^ CCR2/5^+^ cells), with distinction from other monocytic cells based on the co-expression of Col1 and CXCR3 (Fig. 4G, H; Supplementary Fig. 4A, B). These results suggest a significant infiltration of fibrocytes in lung cancer tissue, and increased fibrocyte numbers in the circulation of lung cancer patients.

### Co-injection of fibrocytes with human lung cancer cells augments tumor growth

**Fig. 5:**
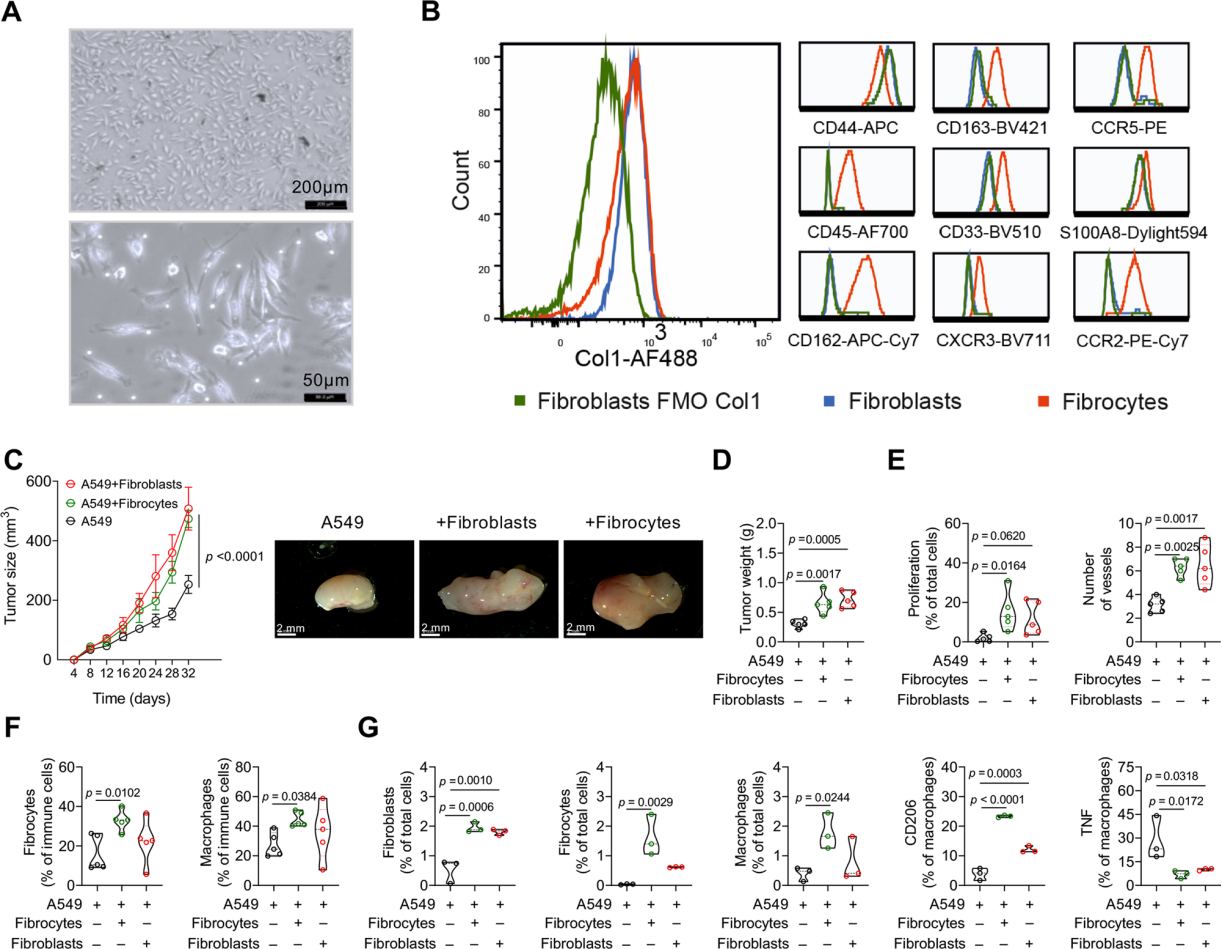
Co-injection of fibrocytes and fibroblasts with lung cancer cells augments tumor growth. Human fibrocytes were isolated from PBMCs. **A** Fibrocytes were depicted by bright field microscopy with different magnifications (*n* = 10, scale bar, 200 µm, and 50 µm). **B** Representative flow cytometry plots show the purity of fibrocytes using CD45^+^, Col1^+^, CD44^+^, CD163^+^ CCR5^+^, CD33^+^, S100A8^+^ CD162^+^, CXCR3^+^, CCR2^+^ as markers compared to fibroblasts. Col1 fluorescence minus one (FMO) control indicates efficacy of the Col1 antibody. **C**–**G** Co-injection of human lung cancer A549 cells with fibrocytes and fibroblasts into BALB/c nude mice. **C** Tumor size from mice injected with A549 (cancer cells) alone or co-injected with fibrocytes and fibroblasts. *p* values were determined using Two-way ANOVA with Fisher’s LSD test, *n* = 5. Representative pictures (scale bar, 2 mm) and (**D**) tumor weight of tumors from mice injected with A549 or co-injected with fibrocytes and fibroblasts after 32 days. *n* = 5, *p*-values were determined using One-way ANOVA with Fisher’s LSD test. **E** Quantification of PCNA^+^ proliferating cells (*n* = 5, 5 images per lung tumor) and vWF^+^ vessels (*n* = 5, 5 images per lung tumor) in tumor tissues from mice injected with A549 or co-injected with fibrocytes and fibroblasts by immunohistochemistry. *p*-values were determined using One-way ANOVA with Fisher’s LSD test. **F** Quantification of fibrocytes (CD45+CD11b+CD162+F4/80+CCR2/5+CD9+CD44+Col1+) and macrophages (CD45^+^CD11b^+^CD162^+^F4/80^+^CCR2/5^–^CD9^–^) in tumors from mice injected with A549 or co-injected with fibrocytes and fibroblasts by FACS analysis, *n* = 5, *p*-values were determined using One-way ANOVA with Fisher’s LSD test. **G** Quantification of fibroblasts (CD45^–^CD163^–^Col1^+^), fibrocytes (CD45^+^CD163^+^CCR2^+^Col1^+^), and macrophages (CD45^+^CD163^+^CCR2^–^Col1^–^), M2-like (CD206^+^) and M1-like (TNF^+^) in tumors from mice injected with A549 or co-injected with fibrocytes and fibroblasts by multiplex immunofluorescence, *n* = 3. *p*-values were determined using One-way ANOVA with Fisher’s LSD test. Source data are provided in the source data file.

As fibrocytes are considered to be the precursors of fibroblasts/myofibroblasts in the lung, we (i) asked whether the identified markers can distinguish human fibrocytes from lung fibroblasts and (ii) investigated the influence of both fibrocytes and fibroblasts on tumor growth using 3 different human lung cancer cell lines. Notably, a panel of our identified fibrocyte markers (CD45, Col1, CD44, CD163, CCR5, CD33, S100A8, CD162, CXCR3, CCR2) clearly distinguished in vitro generated PBMC-derived fibrocytes from primary lung fibroblasts (Fig. 5A, B). To assess the comparative influence of fibrocytes and fibroblasts on tumor growth, we co-injected human fibrocytes or lung fibroblasts together with lung cancer cells into the flanks of BALB/c nude mice. 2 lung adenocarcinoma cell lines, A549 and H1650, and a lung squamous cell carcinoma cell line, H226, were used. Interestingly, fibrocytes promoted A549, H226 and H1650 tumor growth in the co-injected mice, significantly increasing tumor size and final tumor weight (Fig. 5C, D and Supplementary Fig. 5A, B). Moreover, mice co-injected with cancer cells and fibrocytes showed increased cell proliferation (Fig. 5E and Supplementary Fig. 5C) and angiogenesis (Fig. 5E and Supplementary Fig. 5D) as well as enhanced numbers of tumor niche fibrocytes (Fig. 5F, G and Supplementary Fig. 5E, F), macrophages (Fig. 5F, G and Supplementary Fig. 5F, I), fibroblasts (Fig. 5G and Supplementary Fig. 5G) and M2-like macrophages (Fig. 5G and Supplementary Fig. 5J), whereas tumor associated M1-like macrophages were decreased (Fig. 5G and Supplementary Fig. 5K), all compared to mice receiving only cancer cells. Notably, no major differences were observed between fibroblast and fibrocyte co-injection with A549, H226 and H1650 cancer cells, with the exception that only human fibrocyte injection consistently promoted the expansion of mouse fibrocytes and the induction of the M2-like macrophage marker CD206. Taken together, these results provide strong evidence that both, fibrocytes and fibroblasts promote tumor growth by modulating the tumor microenvironment, with a persistent pattern of increased tumor cell proliferation, angiogenesis, and macrophage abundance in A549, H226 and H1650 tumor models, although subtle differences between these two cell types exist.

### Localization of fibrocytes in the lung cancer niche

**Fig. 6:**
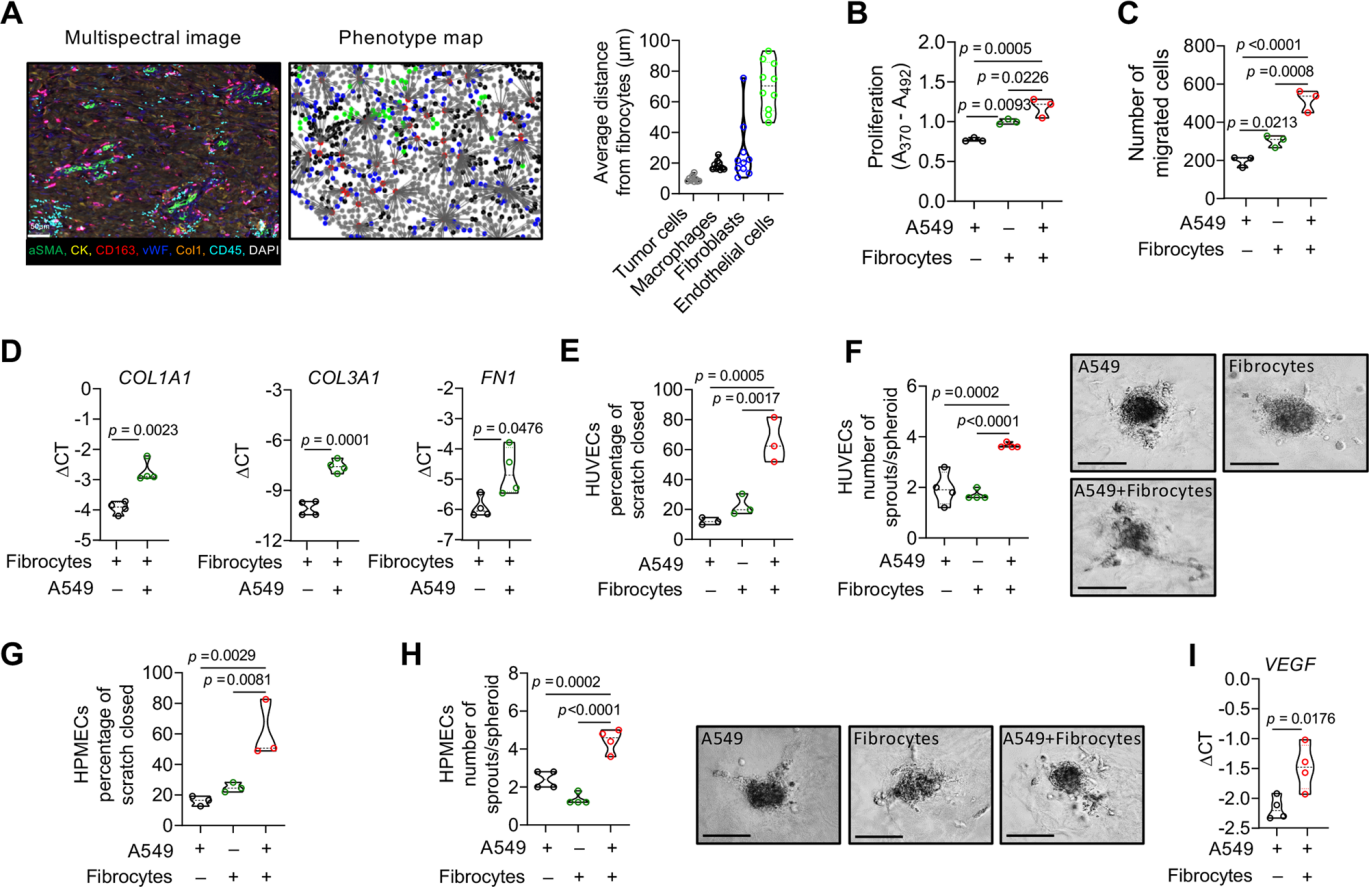
Cross-talk between fibrocytes and cancer cells promotes tumor cell proliferation, migration and endothelial cell sprouting. **A** Representative multispectral image of cells in the human tumor microenvironment labeled with antibodies against pan-cytokeratin (CK), CD45, Col1, CD163, vWF, aSMA, and DAPI, *n* = 10. Colored dots in the cell phenotype map shows, cancer cell (gray, *n* = 52,867), macrophages (block, *n* = 9468), endothelial cells (green, *n* = 1376), fibrocytes (red, *n* = 1174), and fibroblasts (blue, *n* = 5766). Quantification of (**B**) proliferation (*n* = 3 independent experiments, 8 technical replicates) and (**C**) migration (*n* = 3 independent experiments, 3 technical replicates) of A549 in the presence of CM from A549 or fibrocytes or A549 + fibrocyte co-cultures. **D** *COL1A1, COL3A1,* and *FN1* mRNA expression of fibrocytes or A549 + fibrocytes co-cultures, *n* = 4. **E** Quantification of scratch assay in HUVECs in presence of CM from A549 or fibrocytes or A549 + fibrocyte co-cultures (*n* = 3 independent experiments, 3 technical replicates). **F** Quantification (left) and representative pictures (right, scale bar, 130 µm) of sprouting spheroids of HUVECs in the presence of CM from A549 or fibrocytes or A549 + fibrocyte co-cultures (*n* = 4 independent experiments, 5 technical replicates). **G** Quantification of scratch assay in HPMECs in the presence of CM from A549 or fibrocytes or A549 + fibrocyte co-cultures (*n* = 3 independent experiments, 3 technical replicates). **H** Quantification (left) and representative pictures (right, scale bar, 130 µm) of sprouting spheroids of HPMECs in the presence of CM from A549 or fibrocytes or A549 + fibrocyte co-cultures. **I** *VEGF* mRNA expression of A549 or A549 co-cultured with fibrocytes, *n* = 4. **B**, **C**, **E**–**H** *p-*values were determined using One-way ANOVA with Fisher’s LSD test. **D**, **I** *p*-values were determined using Two-tailed unpaired *t*-test with Welsh’s correction Unpaired *t*-test. Source data are provided in the source data file.

The observation that fibrocytes regulate tumor cell proliferation, angiogenesis and macrophage accumulation suggests that fibrocytes reside in a niche, where they directly interact with cancer cells and macrophages. To analyze this in more detail, we explored the spatial orientation of fibrocytes relative to other cells of the lung tumor microenvironment. The analysis of fibrocyte-depletion tumor models and fibrocytes co-injected with cancer cells (FACS analysis and immunohistochemistry) revealed tumor cell proliferation and alteration of macrophage numbers, phenotype and vascularization. Fibroblasts were included since fibrocytes may transdifferentiate into fibroblasts. For the fibrocyte neighborhood measurements, cancer cells, macrophages and endothelial cells were chosen. We performed a multiplex immunolabeling protocol using Opal fluorophores for the following set of markers: Col1, Cytokeratin (CK), CD45, aSMA, vWF, CD163 and DAPI (nuclear stain) (Fig. 6A) to identify cancer cells (Cytokeratin^+^CD45^–^), fibrocytes (CD45^+^Col1^+^CD163^+^), fibroblasts (aSMA^+^Col1^+^CD163^–^), macrophages (CD163^+^ Col1^–^) and endothelial cells (vWF^+^). For the analysis, HALO software was used to extract the fibrocyte neighborhood. The neighborhood was defined as all cells and was characterized by: 1) the total number of fibrocytes, cancer cells, macrophages, fibroblasts, and endothelial cells in the neighborhood; 2) the number of cells in the neighborhood from each of the following phenotypes: cancer cells (CK^+^CD45^–^), fibrocytes (CD163^+^aSMA^-^Col1^+^), endothelial cells (vWF^+^), macrophages (CD163^+^Col1^-^) and fibroblasts (aSMA^+^Col1^+^CD163^-^); 3) as the average distances to fibrocyte of the cellular phenotypes, where the distance was set to 100 μm^29,30^. For the analysis, we selected representative images from 10 tumors, prepared a phenotype map and counted total number of cells per image using DAPI. In our analysis 52,867 cancer cells, 1174 fibrocytes, 1376 endothelial cells, 9468 macrophages and 5766 fibroblasts were used. Neighborhood measurements show that the closest cells were cancer cells (9.5 µm), macrophages (18.4 µm), fibroblasts (26.7 µm), and endothelial cells (70 µm) (Fig. 6A). This suggests that spatial-temporal orchestration of fibrocytes may allow direct interaction with cancer cells, and macrophages to promote tumor development. However, their relative distance to fibroblasts as compared to cancer cells or macrophages suggests a limited potential for fibrocyte to fibroblast trans-differentiation. This notion is supported by the observation that fibrocyte depletion did not alter fibroblasts in tumor models with HSV-TK/Col1 BM chimeras. Thus, we did not further investigate fibrocyte/fibroblast crosstalk.

### Fibrocyte−cancer cell interaction regulates tumor cell behavior, angiogenesis and macrophage phenotype

**Fig. 7:**
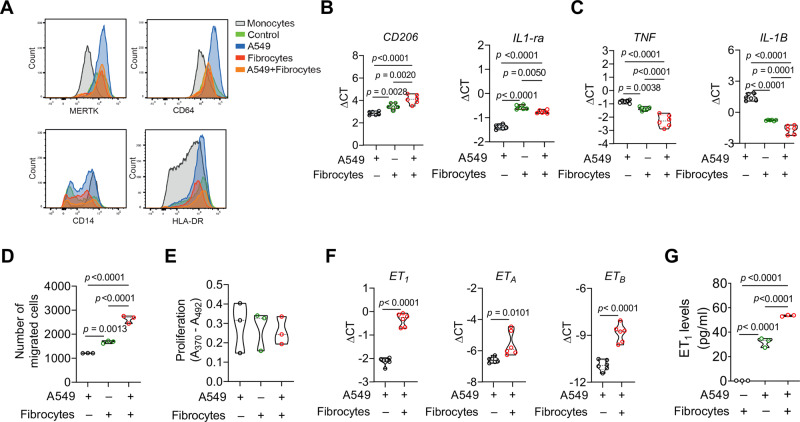
Cross-talk between fibrocytes and cancer cells promotes macrophage activation, migration and endothelial system in cancer cells. **A** FACS analysis of MERTK, CD64, CD14 and HLA-DR cell surface expression on monocytes or monocyte-derived macrophages. The control (green curve) corresponds to macrophages differentiated with human serum. **B**, **C** Expression of macrophage markers *CD206, IL1-ra, TNF*, and *IL-1B* in macrophages incubated with CM from A549 or A549 + fibrocytes co-cultures, *n* = 6. **D** Migration (*n* = 3 independent experiments, 3 technical replicates) and (**E**) proliferation (*n* = 3 independent experiments, 8 technical replicates) of macrophages incubated with CM from A549 or fibrocytes or A549 + fibrocyte co-cultures. **E** *ET*_*1*_*, ET*_*A*_ and *ET*_*B*_ mRNA expression in A549 or A549 + fibrocyte coculture, *n* = 6. **G** Endothelin levels of A549 or fibrocytes or A549 + fibrocytes CM, *n* = 3. **B**–**E**, **G** *P*-values were determined using One-way ANOVA with Fisher’s LSD test. **F** *P*-values were determined using two-tailed unpaired *t*-test with Welsh’s correction. Source data are provided in the source data file.

To determine whether the physical proximity of fibrocytes to cancer cells, endothelial cells or macrophages in the human lung cancer niche may have functional consequences, we generated fibrocytes from human PBMCs (Supplementary Fig. 4A) and co-cultured these fibrocytes with five different lung cancer cell lines (A549, H226, H1650, A427 and HCC15). In all of the cancer cell lines analyzed, conditioned medium (CM) derived from the co-culture of fibrocytes and lung cancer cells resulted in marked increase in cancer cell proliferation and migration in comparison to control cancer cell medium. CM from fibrocytes alone also induced proliferation and migration in various cancer cells, although generally to a lesser extent compared with the co-culture CM (Fig. 6B, C and Supplementary Fig. 6A, B). These observations suggest that the interaction between fibrocytes and cancer cells shifts the cancer cells to a more proliferative and migratory phenotype. Interestingly, we found that communication between fibrocytes and cancer cells altered not only the cancer cell phenotype but also the fibrocyte phenotype toward a more mature connective tissue phenotype with further increased mRNA expression of collagen 1A1 (*COL1A1*) and 3A1 (*COL3A1*) and fibronectin (*FN1*) (Fig. 6D and Figs. S6C–S6F). Next, we assessed the impact of fibrocyte and A549 cell co-culturing on the endothelial cell phenotype using human umbilical vein endothelial cells (HUVECs) and human pulmonary microvascular endothelial cells (HPMECs) (Fig. 6E–H). Notably, CM from fibrocyte and A549 cell co-cultures led to increased migration (scratch assay) and sprouting in HUVECs (Fig. 6E, F), consistent with increased *VEGF* expression in the A549, H226, and H1650 cells undergoing co-culture with fibrocytes (Fig. 6I and Supplementary Fig. 7). To reflect the lung tumor vascularization situation in vitro, we additionally performed co-culture experiments with HPMECs. Importantly, similar to HUVECs, co-culture CM of A549 and fibrocytes led to increased migration (scratch assay) and sprouting (Fig. 6G, H). Moreover, we assessed the impact of fibrocyte and A549 cell co-culturing on the differentiation of monocytes to macrophages and on macrophage phenotype alterations. Interestingly, while CM from fibrocyte and A549 cell co-cultures barely altered monocyte-to-macrophage differentiation (Fig. 7A), it shifted the macrophage phenotype toward M2-like (*CD206* and *IL-1ra*) by reducing M1-like differentiation markers (*TNF* and *IL-1B*) (Fig. 7B, C). In addition, both fibrocyte CM and co-culture CM promoted macrophage migration without affecting macrophage proliferation (Fig. 7D, E). These data suggest that the interaction of fibrocytes with lung cancer cells induces tumor cell proliferation, vessel formation and the accumulation of M2-like macrophages in the lung cancer niche. To identify the chemokines secreted differentially by fibrocytes only, cancer cells only and co-culture, we performed cytokine array from the CM obtained from fibrocytes, cancer cells and co-cultures of cancer cells with fibrocytes. As shown in Supplementary Fig. 8, fibrocytes secrete only IL23 and IFNγ, which were suppressed upon co-culture, whereas cancer cells or fibrocytes secrete CCL5, IL-1ra, CD54, CXCL10, MIF, CXCL1, IL-6, and IL-8, which was not affected by co-culture. Interestingly, Serpin E1 (PAI-1), and CCL2 were induced upon co-culture (Supplementary Fig. 8A–C).

### Fibrocyte−cancer cell interaction regulates the endothelin system in double and triple co-culture systems

Because endothelin-1 (ET_1_) signaling has been implicated in the interaction of fibrocytes with neighboring cells^17^, we investigated the role of this pathway in the fibrocyte−cancer cell interaction. We observed an increase in the expression of mRNAs for endothelin receptor A and B (ET_A_, ET_B_) and ET_1_ in A549 and some of the lung cancer cells and in fibrocytes upon co-culture (Fig. 7F and Supplementary Fig. 9A, B). Further to understand ET_1_ level regulation in different cells and co-cultures, we measured ET_1_ levels using ELISA from fibrocytes, macrophages, cancer cells, endothelial cells (HUVECs) alone and their co-cultures. As shown in Supplementary Fig. 9C the cancer cells and endothelial cells are major producers of ET_1_ compared to fibrocytes. In addition, we observed an increased surface expression of ET_A_ and ET_B_ in co-cultured cancer cells (A549) (Supplementary Fig. 10) and increased ET_1_ levels in co-cultured CM (Fig. 7G). In contrast, the expression of various other chemokines and growth factors previously implicated in cancer biology were not altered neither at the mRNA level (*PDGFA, PDGFB, PDGFC, IGF2, IGF1R, EGF*, and *EGFR;* (Supplementary Fig. 11A–C) nor at the secretome level, except *TGFß1* (Fig. S11D) at mRNA level and Serpin E1 and CCL2 at the secretome level (Supplementary Fig. 8C)*;* under fibrocyte and A549 cell co-culture conditions. Remarkably, treatment with SB-525334, a potent TGFβ inhibitor, showed no effect on mRNA expression level of *ET1, ETA* and *ETB* (Supplementary Fig. 11E) as well as endothelin protein levels (Supplementary Fig. 11F) of A549 cells co-cultured with fibrocytes, suggesting that TGFβ plays no role in regulation of endothelin system.

**Fig. 8:**
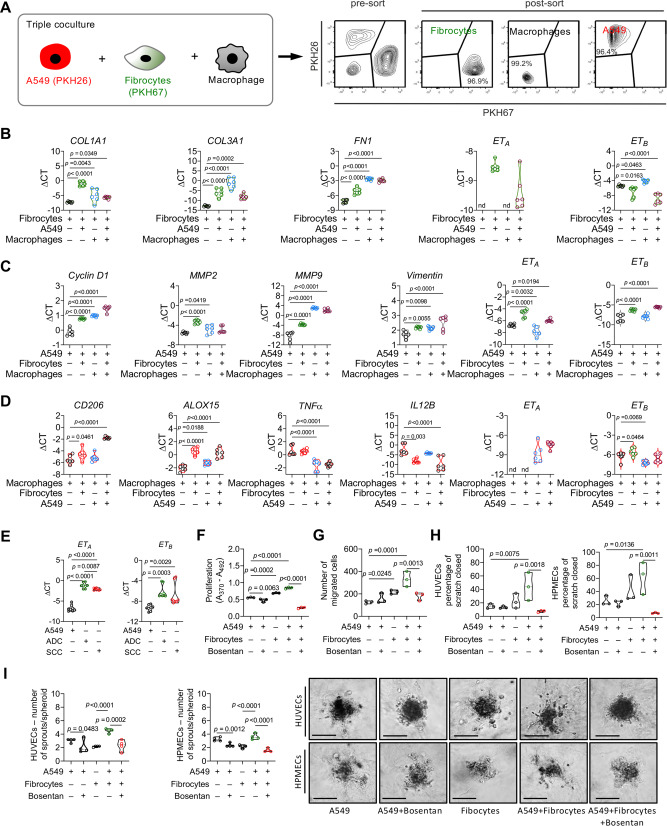
Triple cross-talk among fibrocytes, cancer cells and macrophages affect tumor cell, macrophage and fibroblast phenotypes and Bosentan inhibits tumor cell proliferation and migration, and endothelial cell sprouting. **A** Schematic experimental plan showing fibrocytes and A549 labeled with membrane dyes (PKH26 and PKH67) and co-cultured with unlabeled macrophages. Cell populations were later separated by FACS sorting. **B** mRNA expression of *COL1A1, COL3A1, FN1, ET*_*A*_ and *ET*_*B*_ in fibrocytes co-cultured A549 or macrophages or A549 + macrophages, *n* = 6. **C** mRNA expression of *Cyclin D1, MMP2, MMP9, Vimentin, ET*_*A*_ and *ET*_*B*_ in A549 cells co-cultured fibrocytes or macrophages or fibrocytes+macrophages. **D** mRNA expression of *CD206, ALOX15, TNFα, IL12B, ET*_*A*_ and *ET*_*B*_ in macrophages co-cultured fibrocytes or A549 or fibrocytes or A549 + fibrocytes, n = 6 individual experiments, *n* = 6. **E** mRNA expression of *ET*_*A*_ and *ET*_*B*_ in A549 cells and primary human cancer cells (adenocarcinoma cells, ADC; and squamous cell carcinoma, SCC), *n* = 8. **F**, **G** A549 cells were co-cultured with fibrocytes in the presence and absence of bosentan. **F** Proliferation (*n* = 3 independent experiments, 5 technical replicates) and (**G**) migration (*n* = 3 independent experiments, 3 technical replicates) of A549 cells after incubation with CM from A549 or fibrocytes and fibrocytes+A549 in the presence and absence of bosentan. **H** Scratch assay of HUVECs and HPMECs incubated with CM from A549 cells or fibrocytes or fibrocyte + A549 cells in the presence and absence of bosentan (*n* = 3 independent experiments, 3 technical replicates). **I** Sprouting assay of HUVECs and HPMECs incubated with CM from A549 cells or fibrocytes or fibrocyte + A549 cells in the presence and absence of bosentan (*n* = 4 independent experiments, 5 technical replicates). Representative pictures (scale bar, 100 µm) of sprouting spheroids from HUVEC and HPMECs after incubation with CM from A549 cells compared with fibrocytes and fibrocytes co-cultures in the presence and absence of bosentan. **B**–**I** *p*-values were determined using One-way ANOVA with Fisher’s LSD test. Source data are provided in the source data file.

To mimic the even more complex in vivo situation, we established a triple co-culture system consisting of cancer cells, fibrocytes and macrophages. To distinguish the cell types, we labeled fibrocytes and A549 cancer cells with the fluorescent cell membrane dyes (PKH26 or PKH67), respectively, whereas macrophages remained unlabeled. After co-culturing for 24 h, these cells were sorted by FACS based on membrane dyes or their absence, followed by analysis of mRNAs related to endothelin and its receptors as well as phenotypic markers (Fig. 8A). Each cell type was influenced and phenotypically altered by the triple co-culture conditions, as evidenced by enhanced phenotypic maturation markers such as *COL1A1, COL3A1* and *FN1* in fibrocytes (Fig. 8B); enhanced pro-proliferative and pro-migratory markers such as *Cyclin D1, MMP2, MMP9* and *Vimentin* in cancer cells (Fig. 8C) and enhanced M2-like differentiation markers such as *CD206* and *ALOX15* and decreased M1-like differentiation markers such as *TNF* and *IL12B* in macrophages (Fig. 8D). Interestingly, adding macrophages to a co-culture of fibrocytes and cancer cells suppressed collagen, but not fibronectin expression, even though levels were still higher compared to fibrocytes in monoculture. Notably, we observed an increased expression of ET_A_ levels in all three cell types and upregulation of ET_B_ in cancer cells present in the triple co-culture system (Fig. 8B–D). These data correlated with the increased expression of both *ET*_*A*_ and *ET*_*B*_ in human primary lung cancer cells (Fig. 8E), human lung cancer adenocarcinoma (ADC), lung cancer squamous cell carcinoma tissues (SSC) (Supplementary Fig. 12A) and mouse lung cancer tissues (KRas^LA2^, LLC1) (Supplementary Fig. 12B). Similarly, circulating ET_1_ levels were increased in human lung cancer and altered upon fibrocytes modulation in mouse models of lung cancer (Supplementary Fig. 12C–E).

### Endothelin receptor antagonism attenuates the pro-proliferative and pro-migratory impact of fibrocytes on lung cancer

To probe the functional role of endothelin receptor regulation in the lung cancer niche, we used a dual antagonist of endothelin receptors ET_A_ and ET_B_, bosentan. CM derived from either bosentan-treated fibrocytes and fibrocyte and A549 cell co-cultures caused inhibition of the proliferative and migratory responses of A549 cells, compared with CM from vehicle-treated co-cultures (Fig. 8F, G). Likewise, CM from bosentan-treated fibrocyte and A549 cell co-cultures suppressed the migration (scratch assay) and sprouting abilities of endothelial cells (HUVECs and HPMECs), as compared with CM from vehicle-treated fibrocyte and A549 cell co-cultures (Fig. 8H, I). To unravel underlying molecular mechanisms, we screened for secreted mediators in bosentan or vehicle-treated fibrocyte and A549 cell co-cultures with a cytokine array. We found 25 cytokines (IGFBP-3, IGFBP-2, VEGF, PDGF-AA, IL-6, IL-31 etc.) that were selectively downregulated and 25 cytokines (RANTES, CD40L, TNFα, Cripto-1, etc.) that were selectively upregulated in CM from fibrocyte and A549 cell co-cultures in the presence of bosentan (Supplementary Fig. 13A), indicating a strong impact of endothelin and its receptors on various key factors of the lung cancer microenvironment. Furthermore, stimulating cancer cells with recombinant IGFBP-3, IGFBP-2, VEGF, PDGF-AA or IL-31 led to upregulation of *ET*_*1*_ and *ET*_*A*_ (Fig. S13B). In contrast, blocking individual cytokines (IGFBP-3, IGFBP-2, VEGF, PDGF-AA, IL-6 or IL-31) in A549-fibrocyte co-culture CM using neutralizing antibodies decreased *ET*_*1*_ and *ET*_*A*_ on cancer cells. Although *ET*_*A*_ seemed to be regulated by several of these cytokines, *ET*_*B*_ was mainly regulated by IGFBP-3 and PDGF-AA, Il-6 and IL-31 (Supplementary Fig. 13C). Overall, these results suggest that endothelin receptor antagonism may offer a mechanism for suppression of lung cancer cell proliferation and migration.

### Endothelin receptor antagonism suppresses lung cancer growth in vivo

**Fig. 9:**
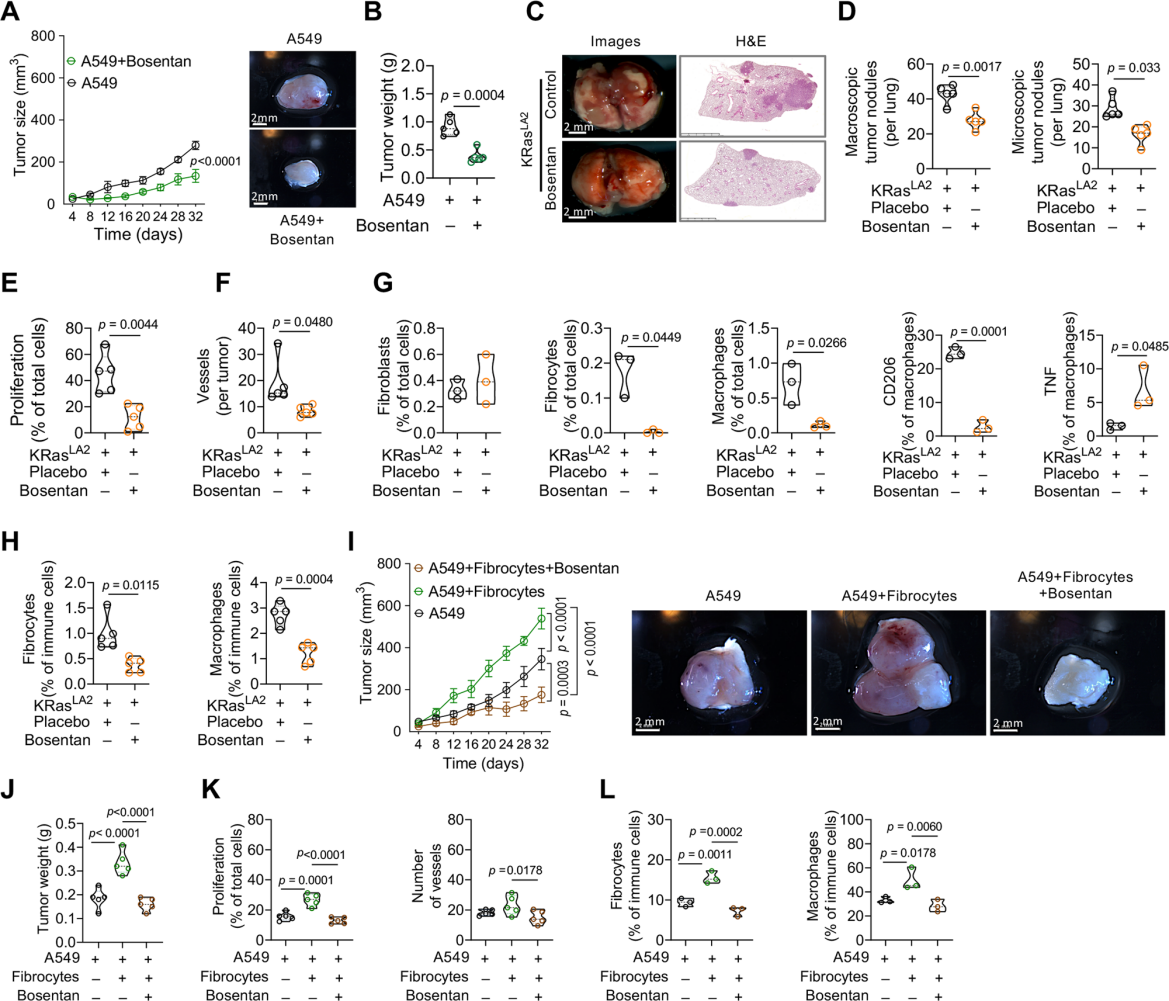
Dual antagonism of ETA and ETB inhibits primary lung tumor growth. **A** Tumor growth curve over 32-days period and tumor images at end of the experiment (scale bar 2 mm) A549 and A549 + bosentan treated tumors, *n* = 5. **B** Tumor weight at day 32, A549 and A549 + bosentan, *n* = 5. **C** Representative photographs (left; scale bar, 2 mm) of whole lungs and H&E stainings of lung sections (right; scale bar, 2.5 mm) in KRas^LA2^ mice with or without (placebo) bosentan treatment, *n* = 5. **D** Quantification of macroscopic and microscopic lung tumor nodules in the KRas^LA2^ mice with or without bosentan treatment, n = 5. Quantification of (**E**) PCNA^+^ proliferating cells (*n* = 5, 8 images per lung) and (**F**) vWF^+^ vessels (*n* = 5, 5 images per lung) in KRas^LA2^ mice with or without bosentan treatment. **G** Quantification of fibroblasts, fibrocytes, macrophages, M2-like and M1-like macrophages with the markers (as described in Fig. 3 legend) in KRas^LA2^ mice with or without bosentan treatment, *n* = 3. **H** Quantification of fibrocytes and macrophages with the markers in KRas^LA2^ mice with or without bosentan treatment, *n* = 5. **I**–**L** A549 or A549 + fibrocytes were subcutaneously (s.c.) injected in BALB/c nude mice in presence or absence of bosentan. **I** Tumor growth curve of A549 or A549 + fibrocytes s.c. tumors in presence or absence of bosentan, *n* = 5. Representative images of the tumors (scale bar, 2 mm). **J** Tumor weight during 32 days, of A549 or A549 + fibrocytes s.c. tumors in presence or absence of bosentan, *n* = 5. Quantification of the (**K**) PCNA^+^ proliferating cells (*n* = 5, 8 images per tumor), CD31^+^ vessels (*n* = 5, 5 images per tumor) in A549 or A549 + fibrocytes tumors in presence or absence of bosentan. **L** Quantification of fibrocytes and macrophages with the markers in A549 or A549 + fibrocytes s.c. tumors in presence or absence of bosentan, *n* = 3. **A**, **I** *p*-values were determined using two-way ANOVA with Bonferroni’s multiple comparison, **B**, **D**–**H** *p*-values were determined using two-tailed unpaired t-test with Welch’s correction and (**J**–**L**) *p*-values were determined using One-way ANOVA with Fisher’s LSD test. Source data are provided in the source data file.

To evaluate the therapeutic potential of endothelin receptor antagonism in vivo, we used three different models of lung cancer: human A549 cells injected into BALB/c nude mice, the KRas^LA2^ oncogenic lung tumor model, and mouse LLC1 cells injected into wild-type mice. Notably, in all models, bosentan treatment significantly suppressed macroscopic and microscopic lung tumor nodules, tumor size and tumor weight as compared with vehicle-treated mice (Fig. 9A–D and Supplementary Fig. 14A, B). Moreover, bosentan treatment reduced cancer cell proliferation and vessel density (Fig. 9E, F) in KRas^LA2^ tumor tissues. Bosentan treatment also resulted in reduced fibrocyte and macrophage accumulation with no alterations in the number of fibroblasts in tumor tissue, and altered M1/M2-like macrophage differentiation profiles (Fig. 9G, H). To further investigate whether the enhancement of lung cancer growth by fibrocytes is also susceptible to endothelin receptor antagonism, we treated BALB/c nude mice that had been injected with A549 cells and fibrocytes with bosentan or vehicle and compared the tumor growth of these mice with that of BALB/c nude mice injected with A549 cells alone. Co-injection of A549 cells and fibrocytes increased the tumor size 2-fold as compared with A549 cell injection alone. Notably, bosentan treatment drastically reduced the tumor size, to a level slightly below that of tumors from mice injected with A549 cells alone (Fig. 9I, J). Furthermore, bosentan treatment in mice injected with A549 cells and fibrocytes reduced cell proliferation, angiogenesis and the number of infiltrating macrophages (Fig. 9K, L). Again, fibrocyte numbers in mice injected with A549 cells and fibrocytes were decreased nearly to the level of mice injected with A549 cells alone when the tumors were treated with bosentan, indicating a major effect of bosentan on fibrocyte proliferation and/or survival (Fig. 9L). Taken together, these results provide strong evidence that fibrocyte-induced promotion of lung cancer growth and of cancer-permissive niche features is almost completely abrogated by endothelin receptor antagonism.

### Anti-tumoral effect of endothelin receptor antagonism requires fibrocytes in vivo

**Fig. 10:**
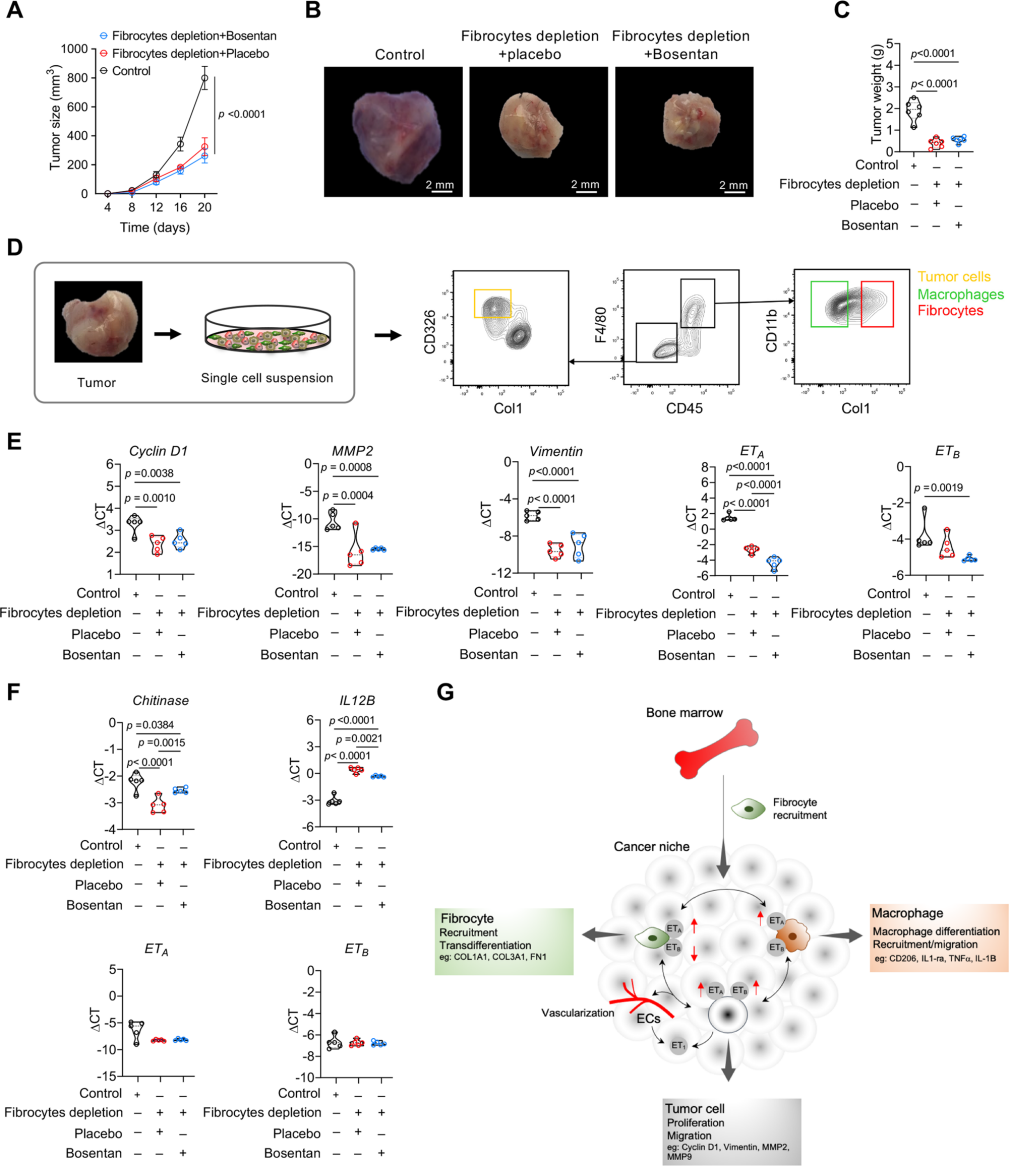
Dual antagonist of ETA and ETB inhibits tumor growth mainly via fibrocytes. **A**–**C** Mice that were transplanted with HSV-TK/Col1 bone marrow were subcutaneously (s.c.) instilled with LLC1 cells and treated with bosentan. **A** Tumor growth curve of control or fibrocyte depleted tumors+placebo and fibrocyte depleted tumors+bosentan during 20 days, *n* = 6. *p*-values were determined using Two-way ANOVA with Bonferroni’s multiple comparison. **B** Representative images of tumor size (scale bar, 2 mm). **C** Tumor weight was measured on day 20 of control or fibrocyte depleted tumors+placebo and fibrocyte depleted tumors + bosentan, *n* = 6. **D** Schematic experimental setup indicating the FACS-sorting strategy to isolate cancer cells, fibrocytes and macrophages from control or fibrocyte depleted tumors + placebo and fibrocyte depleted tumors+bosentan. **E** mRNA expression of *Cyclin D1, MMP2, Vimentin, ET*_*A*_ and *ET*_*B*_ in sorted cancer cells from control or fibrocyte depleted tumors + placebo and fibrocyte depleted tumors + bosentan, *n* = 5. **F** mRNA expression of *Chitinase, IL12B, ET*_*A*_ and *ET*_*B*_ in sorted macrophages from control or fibrocyte depleted tumors + placebo and fibrocyte depleted tumors + bosentan, *n* = 5. **G** Schematic diagram of the mechanism of cross-talk among fibrocytes, cancer cells and macrophages in the lung cancer niche. Increased fibrocyte recruitment and subsequent accumulation in the lung tissues lead to interaction with cancer cells and tumor microenvironmental cells, and activation of the endothelin and its receptors that supports tumor cell growth, immune modulation, and angiogenesis. ET_A_ Endothelin receptor A, ET_B_ Endothelin receptor B, ET_1_ Endothelin 1, COL1A1 Collagen 1A1, COL3A1 Collagen 3A1, FN1 Fibronectin 1, MMP2 Matrix metalloprotease 2, MMP9 Matrix metalloprotease 9, IL-1-ra Interleukin 1 receptor antagonist, TNF-α Tumor necrosis factor-alpha, IL-1B Interleukin-1 beta. **A**, **C**, **E**, **F** *p*-values were determined using One-way ANOVA with Fisher’s LSD test. Source data are provided in the source data file.

In order to evaluate whether fibrocytes were essential for the anti-tumoral effect of bosentan, LLC1 tumor-bearing mice that were transplanted with HSV-TK/Col1 bone marrow were treated with bosentan. Fibrocyte depletion significantly reduced tumor size and weight (Fig. 10A–C). Additional bosentan treatment of these fibrocyte-depleted mice did not further suppress tumor growth (Fig. 10A–C), suggesting that the influence of bosentan on lung tumor growth is largely dependent on fibrocytes. In addition, isolation of cancer cells and macrophages from tumors of fibrocyte-depleted mice with/without bosentan treatment enabled us to investigate molecular alterations in vivo (Fig. 10D). Compared with placebo control, bosentan treatment did not significantly change the mRNA levels of *Cyclin D1, MMP2, Vimentin* and *ET*_*B*_ under conditions of fibrocyte depletion, except leading to a slight downregulation of mRNA expression of *ET*_*A*_ in cancer cells (Fig. 10E). Similarly, bosentan treatment led to no significant changes in the mRNA levels of *Chitinase, IL12B, ET*_*A*_ and *ET*_*B*_ in tumor associated macrophages (TAMs) as compared to TAMs isolated from placebo treated tumors (Fig. 10F). Thus, endothelin receptor antagonism-mediated molecular changes in TAMs and cancer cells and associated reduction of tumor growth require fibrocytes. Along this line, bosentan treatment in naïve (non HSV-TK/Col1 bone marrow transplanted) mice markedly reduced lung tumor growth in three different models of lung cancer: human A549 cells injected into BALB/c nude mice, KRas^LA2^ oncogenic lung tumor model, and mouse LLC1 cells injected into syngenic wild-type mice also in the absence of exogenous fibrocyte co-injection, which may well indicate the impact of bosentan on endogenous mouse fibrocytes.

## Discussion

This study investigated the role of fibrocytes in lung cancer progression and metastasis, with a particular focus on phenotypic switches that happen in the cancer niche. To this end, analysis of human lung cancer tissues and blood samples were combined with co-culturing of human lung cancer cells with various niche cells and genetic ablation, as well as adoptive transfer of fibrocytes in different mouse lung cancer models. Analysis of the fibrocyte-associated molecular interplay in the cancer niche focused particularly on endothelin and its receptors, a potential target for therapeutic intervention.

Fibrocytes were originally identified in mice and humans as monocytic cells expressing hematopoietic stem cell, immune cell and myeloid cell markers, indicating their origin from this lineage^1^. While separating fibrocytes from fibroblasts at the molecular level is simple due to expression of CD45 by the former, differentiating them from monocytes and monocyte-derived cells such as macrophages remained challenging^21^. ScRNA-seq has been frequently used to identify discrete cell populations within bulk samples and therefore appeared to be suitable for uncovering the nature of the elusive fibrocytes. However, one major drawback of scRNA-seq is limited sequencing depth that is not sufficient to identify Col1 expressing cells within the CD45^+^ population in the bone marrow in our and previously published data sets. Vimentin, another putative fibrocyte marker, is expressed by all immune cells, therefore a different strategy was required. We chose to test a two-step protocol, investigating the expression of *Serpinh1* as a marker of collagen-producing cells^20^ and finding associated cell markers, which were then validated in samples where Col1 expressing cells were depleted. Surprisingly, fibrocytes clustered with pDCs, but were separated from these by lack of expression of the pDC marker SiglecH. Since pDC and monocytes develop independently^31^, future studies will be required to delineate the relationship between these cell populations. In mice, fibrocytes were separated from monocytes by expression of mature macrophage markers such as F4/80 and Cd163 and were separated from macrophages by expression of chemokine receptors and Cd9. Similar expression patterns were also found in human samples. Complex lineage-tracing experiments, e.g., by creating Col-1 or SerpinH1 reporter mice will be required to identify the trajectories of fibrocyte development. The six surface marker panel (CD45, CD44, CD162, CD163/F4/80, CD9 and CCR2/5) without the need for intracellular staining of Col-1 suggested by our data may be helpful to guide such studies and to separate fibrocytes from other cell populations in biological samples.

Fibrocytes have been implicated in various chronic inflammatory disorders including those affecting the lung and are suggested to be associated with a poor prognosis^5,7–9^. However, only a single study has quantified fibrocytes in the circulating blood of cancer patients^11^, and the role of this cell type in the pathogenesis of lung cancer is unknown. In the current study, a many fold increase in circulating fibrocytes in patients with lung cancer was noted, and this was paralleled by a marked enrichment of this cell type in the tumor milieu of various subtypes of human lung cancer. Notably, a negative correlation of fibrocyte infiltrate with survival and TNM stages of lung cancer patients support the notion that fibrocytes may be important contributors to the niche organization in lung cancer. An in-depth analysis of the chemotactic mechanisms attracting circulating fibrocytes to the lung cancer microenvironment was not the major focus of the present study, although inflammatory cytokines such as IL-2, TNFα and CXCR4 are implicated in fibrocyte recruitment under different disease conditions^32–35^. This is consistent with preceding observations from our own and other groups that markedly increased levels of cytokines, including IL-2, TNFα and CXCR4, are detected in experimental and human lung cancer tissue^19,24,36–38^. van Deventer et al. reported that fibrocytes predispose the lung to B16-F10 metastasis by recruiting Ly-6C (+) monocytes^16^. Similarly, studies from Afroj et al. demonstrated that fibrocytes derived from PBMCs of patients with lung adenocarcinoma or murine MC38 tumors augmented the proliferation of CD8^+^ T cells with PD-L1 blockade, suggesting the role of fibrocytes in antitumor immunity^39^. In addition, BM-derived fibrocytes were shown to promote stem cell-like properties of lung cancer cells and correlated with high expression of cancer stem cell-associated protein in cancer cells^12^. Clinically, fibrocytes were even suggested to serve as a promising biomarker specifically to predict lung cancer progression after surgery and to mediate the resistance to bevacizumab^40,41^. Although none of the previous studies deeply characterized the fibrocytes with various markers/single cell level (i.e. beyond monocyte marker plus collagen positive) and studied the functional contribution by selectively depleting them using HSV-TK/Col1 mice in the primary lung tumor growth, these studies provided valuable evidence supporting their potential as a biomarker and a role in stemness, antigen-presenting capacity and lung metastasis.

To rigorously probe the contribution of fibrocytes to experimental lung cancer, we generated transgenic mice (HSV-TK/Col1 mice), which, in combination with bone marrow transplantation, allowed for selective depletion of bone marrow−derived fibrocytes. In a subcutaneous transplantation Lewis lung carcinoma model, fibrocyte depletion significantly reduced cancer cell proliferation and tumor size, suppressed formation of the cancer-permissive niche based on reduced angiogenesis and M2-like macrophage numbers, and inhibited metastasis as assessed during tumor relapse. Corresponding results were obtained in KRas^LA2^ lung tumor model. To assess the reverse condition, we co-injected fibrocytes and A549, H226 and H1650 lung cancer cells in an immunodeficient mouse model, which augmented lung tumor growth, in conjunction with enhanced angiogenesis and macrophage abundance in the cancer niche. Thus, these experimental lung cancer models provide strong evidence for a major tumor-supporting role of fibrocytes, most probably by impacting the cancer niche. Comparing the function of fibroblasts and fibrocytes by co-injection studies revealed that both cell types promoted tumor growth. This was not surprising given the frequent observation that fibroblasts are involved in each level of tumorigenesis^42^. However, subtle differences emerged, indicating that fibrocytes more potently skew macrophages towards an M2-like phenotype. Our depletion experiments clearly suggest a non-redundant role of fibrocytes in cancer development. Whether this is due to their impact on tumor-associated macrophages remains to be determined.

For further in-depth analysis of the role of fibrocytes in the cancer microenvironmental “interactome”, the spatial relationship of fibrocytes with other cells of the human lung cancer niche was analyzed, showing the close proximity of fibrocytes with cancer cells, macrophages and vascular cells. Moreover, double and triple co-culturing of fibrocytes with five different types of human lung cancer cells, macrophages and endothelial cells was used to dissect the cellular interplay among these populations. Here we show that the interaction between fibrocytes and cancer cells shifts the cancer cells to a more proliferative and migratory phenotype and, in contrast, shifts the fibrocytes to a more mature connective tissue phenotype. Moreover, in the context of cancer cell−fibrocyte interplay, tube formation and sprouting of endothelial cells was enhanced, and the macrophage phenotype was shifted toward M2-like differentiation phenotype. Thus, essential features of a cancer growth− and metastasis-supportive niche were promoted by the fibrocyte−cancer cell interplay.

Based on the analysis of putative underlying molecular mechanisms, we identified a major role for endothelin and its receptors (ET_1_, ET_A_, ET_B_) in fibrocyte-mediated effects. ET_1_ expression may be linked to VEGF expression and poor prognosis in non-small cell lung carcinoma^43,44^. Moreover, pleiotropic effects of the endothelin system on cancer cells and the host microenvironment have been noted for a number of tumor types^45,46^. Going beyond these previous observations, we suggest that endothelin-mediated fibrocyte-associated mechanisms are essential to promote lung tumor growth and metastasis. Fibrocytes accumulated to a great extent in the lung cancer niche with local upregulation of endothelin and its receptors. Marked upregulation of the endothelin system also occurred in cancer cell−fibrocyte co-cultures with and without macrophages, endothelin receptor antagonism largely blocked the pro-proliferative and pro-migratory impact of fibrocytes on lung cancer cells in vitro and endothelin receptor antagonism suppressed lung cancer growth in vivo. Notably, inhibition of the endothelin receptors interfered with all phenotypic switches that characterize the lung cancer−supportive niche: enhanced proliferation and migration of cancer cells, monocyte-to-macrophage differentiation with M2-like predominance, endothelial migration and tube formation and angiogenesis as well as fibrocyte maturation. Furthermore, by treating LLC1 tumor-bearing mice that were transplanted with HSV-TK/Col1 bone marrow or transplantation-naïve controls with bosentan, we confirmed not only the molecular alterations observed in the co-culture system, but also the fibrocyte-dependency of bosentan’s anti-tumoral effect. Based on these findings, dual ET_A_/ET_B_ blockade might prove to be a treatment concept for lung cancer. However, earlier trials using endothelin receptor antagonist (ERAs) were not successful in pancreatic cancer and metastatic melanoma. Giving ERAs as a monotherapeutic approach or in an advanced stage of disease may explain the therapeutic inefficacy. Thus, clinical trials need to explore the potential use of ERAs in combination therapy regimens, or at an early stage of the disease.

There are some limitations of this study. (i) Inability to detect Col1 in the scRNA-seq performed from the mouse bone marrow and tumor tissues in the current study. This can be due to inherent limitation of the scRNA-seq (i.e. reduced sequencing depth). We circumvented this problem by identifying putative fibrocyte markers based on cells expressing the chaperone for collagen-1 production, SerpinH1, and then validating Col1 expression in these cells by flow cytometry. Indeed, the cells we identified as putative fibrocytes expressed Col1 selectively as indicated by FACS analysis, and were nearly completely depleted by ganciclovir treatment without influencing any other cell populations. Based on above findings, we believe that the fibrocytes we identified express Col1 and are involved in lung cancer progression. (ii) The effects of Col1-TK expressing cells in the KRas^LA2^ model might not be due to exclusive elimination of fibrocytes. Previous publications have elucidated that radiation can influence the KRas-mutant lung cancer^27,28^. Thus, considering the logistic aspects in generating BM chimeras in KRas^LA2^ mouse model and the subsequent influence of irradiation on spontaneous tumor growth in these mice in the presence or absence of Ganciclovir, irradiation experiments in KRas^LA2^ mouse model were not performed. As fibroblasts along with fibrocytes were depleted, we assume that depletion of other Col1 expressing cells (fibroblasts and epithelial cells) might have been involved in reducing tumor growth by Ganciclovir in this model. (iii) Evidence to define whether fibrocytes are the main source of fibroblasts in lung tumor microenvironment. Although current literature suggests that fibrocytes can be a source of fibroblasts in solid tumors^47,48^, our multiplex IHC and FACS analysis of fibroblasts and fibrocytes in various lung experimental set ups rather indicates that fibrocytes are probably not major fibroblast precursors in lung cancer tissues. We believe that they rather maintain their mixed macrophage and extracellular matrix producing nature. However, complex lineage tracing strategies would be required to answer the question if and to what extent fibrocytes contribute to the fibroblast pool in lung cancer.

In conclusion, our results demonstrate a role of fibrocytes in promoting lung tumor growth and metastasis, linked with tumor-supportive phenotypic switches in the cancer niche. Local upregulation of the endothelin system in this niche was demonstrated as a critical mediator of these processes (Fig. 10G), with dual ET_A_/ET_B_ blockade being a potential therapeutic concept.

## Methods

### Animal experiments

Local authorities (Regierungspräsidium Darmstadt, Hessen, Germany) approved all animal studies. All mice were kept under specific-pathogen-free conditions on a 12 h light-dark cycle at 30–70% humidity and temperature of 20–26 °C, with ad libitum access to water and standard chow and handled in accordance with the European Union Commission on Laboratory Animals. C57BL/6 (female and male, age 16 weeks) mice, BALB/c nude mice (CAnN.Cg-Foxn1nu/Crl) (female and male, age 16 weeks) and transgenic lines KRas^LA2^, HSV-TK/Col1 and HSV-TK/Col1 + KRas^LA2^ (female and male, age 12 weeks) were used for these experiments. C57BL/6, immunodeficient BALB/c nude mice (CAnN.Cg-Foxn1nu/Crl) and KRas^LA2^ (female and male, age 12 weeks) mice were purchased from Charles River Laboratories (Sulzfeld, Germany) or Jackson Laboratory (Bar Harbor, ME, USA). HSV-TK/Col1 mice were generated in a C57BL/6 background in our laboratory.

#### Human lung tissues and Human lung specimens

Lung tumor samples from different cancer stages were obtained. Tissue micro arrays (TMAs) were constructed from paraffin blocks of selected lung specimens. For each patient, 1 mm cores from representative areas of the tumor were taken. The Ethical Committee of the University Hospital Munich in Germany approved the collection and analysis of all samples, in accordance with the national laws and the Good Clinical Practice/International Conference on Harmonization guidelines. Informed consent was obtained from all patients. In addition, lung cancer tissue arrays, LUC1501, (Pantomics, Inc., Cat no. LUC 1501; Richmond, CA, USA) and LC20815a (US Biomax, Rockville, USA) were used in this study. All tissues were fixed in 10% neutral buffered formalin for 24 h. Sections were transferred to Superfrost Plus adhesive slides. The tumor specimens were presented as duplicates for internal control and to assess tumor heterogeneity. The study protocol for tissue donation (donor lung tissue^49^ and lung tumor tissue) was approved by the ethics committees of the University Hospital Giessen (Giessen, Germany) and University Hospital Munich (Munich, Germany) in accordance with national law and with “Good Clinical Practice/International Conference on Harmonisation” guidelines. Written informed consent was obtained from each patient^50^.

#### Single-cell RNA sequencing (scRNA-seq) sample preparation and data analysis

Bone marrow derived cells were isolated from C57BL/6 mice (healthy) tibia and femur using an aseptic technique. The bone marrow was flushed out of the bone marrow cavity by using a 26-gauge needle with 10 ml PBS. Subsequently cell suspension was passed through a 70 µm nylon mesh. After a 10 min centrifugation step at 500 x g, cells were incubated in 2 ml Erylysis buffer for red blood cell removal. Lysis was stopped after 4 min incubation time, by adding 23 ml PBS. For LLC1 or KRasLA2 tumor bearing mice CD45 cell isolation. Briefly, lungs were minced into small pieces and treated with Collagenase 5% and DNAase 1% containing buffer for 30 min at 37 °C. Afterwards cells were passed through a 100 µM cell strainer followed by 40 µm nylon mesh. After a 10 min centrifugation step at 500 x g, cells were incubated in 2 ml Erylysis buffer for red blood cell removal. After 4 min incubation time, lysis was stopped by adding 23 ml PBS. Cells were resuspended in PBS and FACS sorted for CD45 cells using an AriaIII-sorter and 7-AAD as indicator for dead cells. The cell suspensions were counted with Moxi cell counter and diluted according to manufacturer’s protocol to obtain 10.000 single cell data points per sample. Each sample was run separately on a lane in Chromium controller with Chromium Next GEM Single Cell 3**ʹ** Reagent Kits v3.1 (10xGenomics). Single cell RNAseq library preparation was done using standard protocol. Sequencing was done on Nextseq500 and raw reads were aligned against the mouse genome (mm10) and counted by StarSolo v2.7.3a^51^ followed by secondary analysis in Annotated Data Format. Preprocessed counts were further analyzed using Scanpy v1.6^52^. Basic cell quality control was conducted by taking the number of detected genes and mitochondrial content into consideration. We removed 214 cells in total that did not express more than 200 genes or had a mitochondrial content less than 3%. Further, we filtered genes if they were detected in less than 30 cells (<0.3%). Raw counts per cell were normalized to the median count over all cells and transformed into log space to stabilize variance (accession number GSE179191). We initially reduced dimensionality of the dataset using PCA, retaining 50 principal components. Subsequent steps, like low-dimensional UMAP embedding (McInnes & Healy, https://arxiv.org/abs/1802.03426) and cell clustering via community detection (Traag et al. https://arxiv.org/abs/1810.08473), were based on the initial PCA. Final data visualization was done by cellxgene v1.0.0 package.

#### Cell culture

Tumor cell lines, including A549 (CCL-185), A427 (HTB-53), H1650 (CRL-5883), H226 (CRL-5826) and LLC1 (CRL-1642) were obtained from American Type Culture Collection, HCC15 (ACC 496) cells were purchased from DSMZ (Braunschweig, Germany). Cell lines were cultured according to the manufacturer’s recommendations. A549 cells were cultured in Dulbecco’s Modified Eagle Medium (DMEM; Gibco-BRL, Karlsruhe, Germany) supplemented with 10% fetal calf serum (FCS; Gibco) and 1% penicillin/streptomycin (P/S). A427, H1650, H226, HCC15 and LLC1 cells were cultured in Roswell Park Memorial Institute (RPMI) 1640 medium (Gibco) supplemented with 10% FCS and 1% P/S. HUVECs (PB-CH-190-8013) were obtained from PELOBiotech GmbH and cultured in EGM™−2 Bulletkit™ (Lonza Group AG, Basel, Switzerland) supplemented with 2% FCS and VEGF until passage seven. HPMEC (C-12281) were obtained from PromoCell (Heidelberg, Germany) and cultured in Endothelial Cell Growth Medium MV with supplements (# C-22220, # C-39220, Sigma-Aldrich, Taufkirchen, Germany).

#### Generation of fibrocytes

Peripheral blood mononuclear cells (PBMCs) were isolated by density-gradient centrifugation over Ficoll-Paque (GE Healthcare Bio-Sciences AB, Freiburg, Germany) according to the manufacturer’s instructions, followed by washes to remove platelets and resuspended in fibrocyte medium, which consisted of DMEM with low glucose (1 g/L) and pyruvate (Gibco Life Technologies) supplemented with 1% P/S and 20% FCS. All cell cultures were performed at 37 °C, 5% CO_2_ in a humidified incubator. After 3 days, non-adherent cells were removed and the adherent cells were incubated in complete DMEM for additional 10 days. On the fifth day, 50% of the conditioned medium (CM) was replaced with fresh medium. Fibrocytes were negatively selected using the Dynabead method by incubation with antibodies against T cells, monocytes and B-cells CD2 (Merck #MABF83, clone RPA-2.10) 1 µg/µl; CD14 (Merck #MAB1219, clone 2D-15C) 0,5 µg/µl; CD19 (Merck #MAB1794, clone FMC63) 0,1 µg/µl for 40 min at 4 °C. Dynabeads Pan Mouse IgG (Invitrogen/Life Technologies Carlsbad, USA) was added to the cells at a concentration of more than five beads per target cell. Cells were incubated for an additional 40 min, followed by being placed in a magnetic holder to remove the antibody-labeled cells. Then, the remaining fibrocytes were reseeded into 6-well tissue culture plates for further experiments such as co-culturing with cancer cells.

#### Generation of macrophages

PBMC-derived monocytes were cultured in RPMI medium supplemented with l-glutamine and 1% P/S and were seeded in poly-d-lysine−coated culture dishes (Thermo Fisher Scientific, Waltham, USA). After 1 h, the medium was replaced with RPMI medium supplemented with l-glutamine, 2% human serum and 1% P/S. Cells were cultured for 10 days with replacement of the medium every other day until undifferentiated macrophages were obtained.

#### Co-culture of fibrocytes with cancer cells and macrophages

A549 cells were first seeded on opposite sides of 8 µm-pore Transwell filter inserts (3 × 10^5^ cells/well; BD BioSciences, Heidelberg, Germany) for 2 h prior to culturing in 6-well companion plates (BD Biosciences) for 24 h. The A549 cells were then co-cultured with the fibrocytes at a ratio of 1:1 for 24 h, followed by treatment with the inhibitors bosentan (200 µM; Selleckchem, Houston, USA), the concentrations of which were selected as described^53,54^. The same co-culture ratio was also used for co-culturing fibrocytes with A427, H1650, H226 and HCC15. Regarding fibrocyte, A549 cell and macrophage triple co-cultures (at a ratio of 1:1:1), fibrocytes were labeled with PKH26 (red fluorescence; Sigma) and A549 cells were labeled with PKH67 (green fluorescence; Sigma), whereas macrophages were unlabeled prior to co-culturing for 24 h. Triple co-cultures were separated using FACS sorting (FACSAria III Cell Sorter). Control cultures consisted of groups treated with the same amount of DMSO (the vehicle for bosentan). To avoid effects from residual bosentan and DMSO in the medium, the cells were washed three time with PBS after treatment, followed by an additional culturing period of 24 h with fresh medium. Next, the conditioned medium (CM) was collected and centrifuged at 1000 rpm for 5 min and was stored at −80 °C until used for subsequent experiments for downstream analyses.

#### Proliferation assay

The effect of CM on the proliferation of lung cancer cell lines (A549, H226, HCC15, H1650 and A427) was determined by using a colorimetric cell proliferation ELISA BrdU kit (Roche Applied Science, Penzberg, Germany) according to manufactures instructions. Briefly, cells were seeded in 96-well plates with 1 × 10^4^ cells/well and cultured overnight. The medium was then replaced with serum-free medium, and the cells were serum starved for 24 h, followed by a 24 h treatment with CM. Then BrdU was added to the cells for 2 h, and BrdU incorporation was detected using horseradish peroxidase (HRP)-conjugated antibody by measuring the absorption of the HRP substrate at 370 nm with reference wave length at 492 nm using a Tecan Infinite M200 PRO plate reader.

#### Migration assay

Chemotactic migration was quantified using a Boyden chamber Transwell assay. Cancer cells (A549, H226, HCC15, H1650 and A427) or PBMC-derived macrophages were seeded in the upper part of a chamber (8.0 μm pore size; BD Biosciences) at a density of 0.05 × 10^6^ cells/chamber in 300 µl DMEM. CM (700 µl**/**well) was distributed to each well of the 24-well companion plates (BD BioSciences). Serum-free and serum-rich media were used as migration controls. Cells were incubated for 8 h at 37 °C. Then the chambers were washed with PBS and placed in methanol for fixation, followed by 10 min staining with crystal violet. After a final wash with H_2_O, each membrane was mounted on a slide with Pertex (Medite GmbH, Burgdorf, Switzerland), slides were scanned with a Nanozoomer 2.0HT digital slide scanner C9600 (Hamamatsu Photonics). The number of migrated cells per membrane was counted using ImageJ software.

#### Flow cytometry

A single-cell suspension from tumor tissue samples was obtained by using the mouse Tumor Dissociation kit (Miltenyi Biotec, CA, USA). PBMCs from healthy individuals and from patients with lung cancer were isolated by density-gradient centrifugation over Ficoll-Paque and were then used to assess the number of circulating fibrocytes. Fluorochrome-conjugated primary antibodies were added to single-cell suspensions and incubated for 30 min. Antibodies for staining murine bone marrow and tumor samples were: B220-APC/Fire 750 (Biolegend #103260, RA3-6B2: 1:100), CCR2-PE-Cy7 (Biolegend #150612, SA203G11, 1:100), CCR5-PE-Cy7 (Biolegend #107018, HM-CCR5, 1:50), CXCR3-BV711 (BD Biosciences #740825, CXCR3-173, 1:100), CD9-BV650 (BD Biosciences #564236, KMC8, 1:100), CD11b-BV605 (BD Biosciences #563015, M1/70, 1:200), CD44-AlexaFluor 700 (BD Biosciences # 560567, IM7, 1:100), CD45-Vioblue (Miltenyi Biotec #130-118-953, 30F11, 1:50), CD162-BV510 (BD Biosciences #563448, 2PH1, 1:200), CD326/Epcam-BV711 (BD Biosciences #563134, G8.8, 1:100), F4/80-PE-Cy7 (Biolegend # 123114, BM8, 1:100), Ly-6C-PerCP-Cy5.5 (Biolegend, AL-21, 1:200), Ly-6G-APC-Cy7 (Biolegend #127624, 1A8, 1:100), RANKL-AlexaFluor 647 (BD Biosciences #560296, IK22-5, 1:50), Sca-1-PE-CF594 (BD Biosciences # 562730, D7, 1:100), Siglec H-APC (Biolegend # 129612, 551, 1:50). Antibodies used for staining human PBMCs, fibrocytes, fibroblasts and macrophages were CCR2-PE-Cy7 (Biolegend #357212, K036C2,1:50), CCR5-PE (Biolegend #359106, J418F1, 1:50), CXCR3-BV711 (BD Biosciences #563156, 1C6, 1:100), CD14-APC H7 (BD Biosciences #560180, Møp9), CD33-BV510 (BD Biosciences #563257, WM53), CD44-APC (Biolegend #338806, BJ18,1:200), CD45-AlexaFluor 700 (BD Biosciences #560566, HI30, 1:50), CD64-BV605 (BD Biosciences #740406, 10.1, 1:100), CD162-APC/Fire 750 (Biolegend #328814, KPL-1, 1:100), CD163-BV421 (BD Biosciences #566277, GHI/61, 1:100), CD163-FITC (Biolegend #333618, GHI/61, 1:100), HLA-DR-APC (Biolegend #980406, L243, 1:200) and MERTK-BV421 (Biolegend #367604, 590H11G1E3, 1:50). Expression of endothelin receptors on cells from co-cultures was determined using EDNRA-AlexaFluor 405 (R&D Systems, 485709, 1:50) and EDNRB-AlexaFluor 750 (R&D Systems,485709, 1:50) antibodies. Intracellular human and mouse collagen I was stained with polyclonal Collagen-I-FITC antibodies (ThermoFisher Scientific, #600-402-103, 1:100), anti-human S100A8 with S100A8-DyLight 594 antibody (Novus Biologicals, 63N13G5, 1:200), after fixation and permeabilization using a Fixation/Permeabilization Solution Kit from BD Biosciences. The flow cytometric analysis was performed using a LSRII/Fortessa flow cytometer, and the data were acquired using FACSDiva software v9.0 and analyzed with FlowJo_v10 (TreeStar) software. All antibodies and secondary reagents were titrated to determine optimal concentrations. CompBeads (BD Biosciences) were used for single color compensation to create multi-color compensation matrices. For gating, fluorescence minus one control were used. The instrument was calibrated using Cytometer Setup and Tracking beads (BD Biosciences). To maintain the integrity of the RNA in PFA-fixed cells, blocking and antibody labeling was performed in the presence of high salt buffer (2 M NaCl)^55^.

#### Cytokine array

The human cytokine antibody array panel A and human xl cytokine array kit (R&D Systems) was used to detect secreted mediators in CM. Nitrocellulose membranes spotted with capture 36 or 105 antibodies that target specific cytokines were first blocked and subsequently incubated with CM. The antibodies-cytokine-antibodies coupling reaction was detected by adding a streptavidin-HRP solution, followed by incubation with Chemi Reagent Mix. The reactions were visualized with Amersham Hyperfilm ECL films (GE Healthcare Europe GmbH). Analysis and quantification were performed with the BioDoc Analyze Software 2.67.5.0 (www.biometra.com).

#### Quantitative real-time PCR (qPCR)

Total RNA was extracted by using an miRNeasy Micro kit (Qiagen). For the isolation of RNA from cells obtained after FACS sorting of tumor tissue, RNA was isolated using Qiagen’s FFPE kit according to the manufacturer’s protocol. For all samples the highest recommended volume of PKD buffer (240 μl) was used to avoid interference from remnants of high salt buffer. cDNA reverse transcription from 800 ng of total RNA was performed using the ImPromII^TM^ Reverse Transcription System (Promega, Madison, USA). Real-time RT-PCR was performed with Brilliant III Ultra-Fast SYBR® Green QPCR Master Mix (Agilent Technologies) using StepOne™ and StepOnePlu ™Software v2.3 for qRT-PCR. The data were normalized to the expression of the housekeeping gene hypoxanthine phosphoribosyltransferase1 (HPRT). Human gene-specific primers were used as follows: *HPRT* forward 5′-TGACACTGGCAAAACAATGCA-3′ and reverse 5′GGTCCTTTTCACCAGCAAGCT-3′; *ET*_*1*_ forward 5′-GGCTGAAGGATCGCTTTGA-3′ and reverse 5′-TAAGACTGCTGTTTCTGGAGC-3′; *ET*_*A*_ forward 5′- TGCCCTTGGAGACCTTATCTA-3′ and reverse 5′-CAACTGCTCTGTACCTGTCA-3′; *ET*_*B*_ forward 5′- GCTACACTGTCTGGCATTCT-3′ and reverse 5′- CAGTTTCAGAGCCTCTCGG-3′; *VEGF* forward 5′-AGGGCAGAATCATCACGAAGT-3′ and reverse 5′-GGTCTCGATTGGATGGCAGTA-3′; *TNFα* forward 5′-GAGGCCAAGCCCTGGTATG-3′ and reverse 5′-CGGGCCGATTGATCTCAGC-3′; *IL12B* forward 5′-GCCCAGAGCAAGATGTGTCA-3′ and reverse 5′-CACCATTTCTCCAGGGGCAT-3′; *IL1B* forward 5′-AGAAACTGGCAGATACCAAACC-3′ and reverse 5′-TGGAAGGAGCACTTCATCTGT-3′; IL-*1ra* forward 5′-CTATGAGGCCCTCCCCATGGC −3′ and reverse 5′-CAACTAGTTGGTTGTTCCTCC-3′; *CD206* forward 5′-ACAACAAAAGCTGACACAAGGA-3′ and reverse 5′AGGACAGACCAGTACAATTCAG-3′; *ALOX15* forward 5′-CTTCAAGCTTATAATTCCCCAC-3′ and reverse 5′-GATTCCTTCCACATACCGATAG-3′; *Cyclin D1* forward 5′- TGCCACAGATGTGAAGTTCATT-3′ and reverse 5′-GGACAGGAAGTTGTTGGGG-3′; *Vimentin* forward 5′-GGAAATGGCTCGTCACCTTCGT-3′ and reverse 5′- GCAGAGAAATCCTGCTCTCCTCG-3′; *MMP2* forward 5′-ACCAGCTGGCCTAGTGATGA-3′ and reverse 5′-TTCAGGTATTGCATGTGCTAGGT-3′; *MMP9* forward 5′-CGGAGCACGGAGACGGGTAT-3′ and reverse 5′-GAGTTGGAACCACGACGCCC-3′; *COL1A1* forward 5′-AAGCGAGGAGCTCGAGGTGAAC-3′ and reverse 5′-TTGGCACCAGGCAGACCAGCTT-3′; *COL3A1* forward 5′-TGGGAGAAATGGTGACCCTGG-3′ and reverse 5′-CCAGGATAGCCTGCGAGTCCT-3′; *FN1* forward 5′-CATGAAGGGGGTCAGTCCTA-3′ and reverse 5′-CTTCTCAGCTATGGGCTTGC-3′; *PDGFA* forward 5′-GAGGACCTTGGCTTGCCTGC-3′ and reverse 5′-GGCCAGCCTCTCGATCACCT-3′; *PDGFB* forward 5′-TCTCTGCTGCTACCTGCGT-3′ and reverse 5′-CAAAGGAGCGGATCGAGTGG-3′; *PDGFC* forward 5′-TAGGGCGCTGGTGTGGTTCT-3′ and reverse 5′-GTTGTAGTGGATGCAGAACCCTGG-3′; *EGF* forward 5′-CGCCCTAAGTCGAGACCGGAA-3′ and reverse 5′-TCCTACAGGGCACGTGCAGTAA-3′; *EGFR* forward 5′-GCGTTCGGCACGGTGTATAA-3′ and reverse 5′-GCTTTCGGAGATGTTGCTTC-3′; *IGF2* forward 5′-CCTCCAGTTCGTCTGTGGG-3′ and reverse 5′-CACGTCCCTCTCGGACTTG-3′; *IGF1R* forward 5′-CGTGCGCTGGATGTCTCCTG-3′ and reverse 5′-AGAAGGCCGCCCTCCATGAC-3′; *TGFß1* forward 5´- GCGTGCGGCAGCTGTACATT-3´ and reverse 5´- GGGCCAGGACCTTGCTGTACT-3´; Mouse gene-specific primers were as follows: *Hprt* forward 5’ GCTGACCTGCTGGATTACAT 3’ and reverse 5’ TTGGGGCTGTACTGCTTAAC 3’; *Cyclin D1* forward 5’ TCAAGTGTGACCCGGACTG 3’ and reverse 5’ ATGTCCACATCTCGCACGTC 3’; *Mmp2* forward 5’ GGTGGTGGTCATAGCTACTTCT 3’ and reverse 5’ TCACATCCTTCACCTGGTGTG 3’; *Vimentin* forward 5’ GATGGACAGGTGATCAATGAGA 3’ and reverse 5’ TGTTAAGTGCTGAGCTTCTTTCT 3’; *Chitinase* forward 5’ GAGTGCTGATCTCAATGTGGATT 3’ and reverse 5’ GGGTCACTCAGGGTAAAGGT 3’; *Il12b* forward 5’ AGCACGGCAGCAGAATAAAT 3’ and reverse 5’ GTCTGGTTTGATGATGTCCCT 3’; *ET*_*A*_ forward 5’ CACCTCAAACAGCGTCGAGA 3’ and reverse 5’ TGCCAGGTTAATGCCGATGT 3’; *ET*_*B*_ forward 5’ AACACCTACAAGTTGCTCGC 3’ and reverse 5’ TGTGATTCCCACAGAAGCCT 3’.

#### Culturing of primary cancer cells and fibroblasts

The University of Giessen Biobank provided cancer cells isolated from human lung tumors (NSCLC). Cells were grown in DMEM F12 (supplemented with sodium selenite, ethanolamine, phosphoryl ethanolamine, sodium pyruvate, adenine and HEPES) and maintained for a maximum of 7–8 passages.

Primary human lung fibroblasts were derived from healthy donor lung tissue and cultured in MCDB 131 medium (Gibco, 10372-019) supplemented with 5% FCS, 1% penicillin/streptomycin, 1% Glutamine, 5 µg/mL Insulin, 2 ng/mL bFGF and 0.5 ng/mL EGF^56^.

#### Cytokine treatment

Human recombinant cytokines IL-31 (30 ng/mL, Peprotech, 200-31), IL-6 (200 ng/mL, Peprotech, 200-06), PDGF-AA (20 ng/mL, Peprotech, 100-13 A), VEGF (40 ng/mL, Peprotech, 100-20), IGFBP-2 (40 ng/mL, BioLegend, 750302) and IGFBP-3 (40 ng/mL, BioLegend, 555602) were administrated to A549 cells individually and incubated for 24 h. Then RNA of treated A549 was isolated and reverse transcribed to cDNA for detection of *ET*_*1*_*, ET*_*A,*_ and *ET*_*B*_ expression using qRT-PCR.

#### Neutralizing antibody treatment

Neutralizing antibodies: IL-31 (2 μg/mL R&D Systems #AF2824), IL-6 (1 μg/mL R&D Systems #MAB2061-100), PDGF-AA (2 μg/mL R&D Systems #AB-221-NA), VEGF (0.1 μg/mL R&D Systems #MAB293-100), IGFBP-2 (2 μg/mL R&D Systems #AF674), and IGFBP-3 (1 μg/mL R&D Systems #AF675) were depleted individually from conditioned medium of A549-fibrocyte co-culture using neutralizing antibodies. Neutralizing antibodies or control IgG were preincubated for 1 h with conditioned medium before administration to A549 cells for a period of 24 h incubation. Then RNA of treated A549 was isolated and reverse transcribed to cDNA for detection of *ET*_*1*_*, ET*_*A*_ and *ET*_*B*_ expression using qRT-PCR.

### Injection and co-injection of cells

Mouse LLC1 cells (1 × 10^6^) were injected into male C57BL/6 mice subcutaneously (s.c.). At day 16, mice were sacrificed and their tumors were harvested and immersed in formalin for paraffin sections or in Tissue Tek for cryosections and histologically examined. Human adenocarcinoma A549 cells (3 × 10^6^) were injected into BALB/c nude mice s.c. In both mouse models, tumor volume based on caliper measurements was calculated by the modified ellipsoidal formula (L × W^2^)/2, the maximal tumor size of 1500 mm^3^ was not exceeded. For co-injection experiments, A549 cells, H226 cells, and H1650 cells either alone (3 × 10^6^ cells per 200 µl NaCl) or together with fibrocytes (3 × 10^6^ cancer cells or 0.6 × 10^6^ fibrocytes or 0.6 × 10^6^ fibroblasts per 200 µl NaCl; ratio, 5:1) were injected s.c. into the hind flank of BALB/c nude mice. At day 20 (LLC1), day 32 (A549) or day 16 (H226, H1650), mice were sacrificed and their tumors were harvested, immersed in formalin for paraffin sections or in Tissue Tek for cryosections and were histologically examined^19,57^.

### Tumor relapse model

Primary tumor growth was initiated as described above in C57BL/6 mice. After 10 days, the primary s.c. tumor was extracted and the wound sutured. All mice were intensively observed over the following 20−32 days. Mice were sacrificed using predetermined humane endpoints, and their lungs (after perfusion) were extracted. The organs were photographed, macroscopic nodules were quantified and the tissue was fixed in 4% paraformaldehyde for immunohistochemistry^19^. Part of the lung was used for FACS analysis.

### Intravenous injection of cancer cells

LLC1 cells (1 × 10^6^) were injected into C57BL/6 mice intravenously by the tail vein. All mice were intensively observed over the following 18 days. Mice were sacrificed using predetermined humane endpoints, and their lungs (after perfusion), were extracted. The lungs were photographed, macroscopic nodules were quantified and the tissue was fixed in 4% paraformaldehyde for immunohistochemistry^25^. Part of the lung was used for FACS analysis.

### HSV-TK/Col1

HSV-TK/Col1 mice were generated as described^22^. Briefly, purified pCol1-TK/IRES-EGFP plasmids were diluted in injection buffer. Fertilized embryos for the microinjection were obtained from the oviducts of super-ovulated female mice immediately after detection of a vaginal plug. The purified plasmid was microinjected into the pronucleus of a fertilized ovum, and groups of injected embryos were re-implanted into the oviducts of pseudo-pregnant female mice. Once litters were delivered, their tail tissues were analyzed for genotyping, and positive mice were bred with wild-type C57BL/6 mice. Offspring from the second round of breeding were used to establish an HSV-TK double-positive mouse colony, and these mice were used for experiments^22^.

#### Bone marrow isolation and transplantation

Bone marrow was isolated from donor HSV-TK positive mice for transplantation into C57BL/6 recipient mice. Donor mice were injected intraperitoneally with 500 Units of heparin (Heparin-Natrium Braun 25000 I.E./5 ml, B. Braun Melsungen AG, Germany) and were anesthetized with a lethal dose of 10% ketamine (Bela-pharm GmbH & Co. KG, Vechta, Germany), 2% xylazine (Rompun®, Bayer Vital GmbH, Leverkusen, Germany) and 0.9% sodium chloride (NaCl) (B. Braun Melsungen AG, Melsungen, Germany) at a ratio 1:1:2. femurs were then dissected free of muscle tissues. The bones were flushed with culture medium with 1% Penicillin/Streptomycin until all bone marrow cells were out. Cells were centrifuged at 800 rpm for 7 min at 4 ^o^C, resuspended in fresh medium and filtered through a cell strainer (40 µm pore size). The cells were then counted and transplanted into acceptor mice that had undergone irradiation (3 × 10^6^ cells/100 µl NaCl per mouse injected intravenously).

### KRas^LA2^

We used a mouse model derived from spontaneous somatic activation of the latent KRas^LA2^ allele, which develops alveolar adenomatous hyperplasia 1 week after birth^26^.

### HSV-TK/Col1 + KRas^LA2^

HSV-TK/Col1 mice were crossed with the KRas^LA2^ mice. The double transgenic mice (12 weeks old) were treated with ganciclovir for 8 weeks to deplete fibrocytes as described^22^.

#### Ganciclovir treatment and LLC1 cancer cells injection

C57BL/6 mice were given ganciclovir (Cymevene®, Roche Holding GmbH, Germany) via their drinking water at a concentration of 4 mg/ml to eliminate all bone marrow derived circulating fibrocytes^22^. Pre-treatment continued for 5 days after which this LLC1 cells were injected into syngeneic C57BL/6 mice as described above. Application of ganciclovir continued throughout the whole of experiment.

#### Bosentan treatment

For inhibitor studies, the dual endothelin inhibitor bosentan (10 mg/kg body weight; Sigma) dissolved in DMSO or DMSO alone (vehicle control) was applied daily intraperitoneally.

#### Single-cell isolation

Mice were sacrificed and their tumors removed. The tumors were then diced, and the tissue was digested with 5 µg/µl collagenase supplemented with 10 µg/µl DNase at 37 °C for 30 min. After the mixture was passed through cell strainers along with red blood cell lysis, cells were resuspended in PBS until further use^24^.

#### Hematoxylin and eosin staining

Tissue sections were deparaffinized in xylene and then rehydrated sequentially in 100, 90, and 70% ethanol in distilled water. The slides were then incubated in fresh hematoxylin (Invitrogen Corporation, Frederick, MD, USA) for 20 min, washed in distilled water, incubated in acidified eosin solution (Richard-Allan Scientific, Kalamazoo, MI, USA) for 4 min and then washed in distilled water. Finally, the slides were dehydrated in 90 and 100% ethanol, air dried and mounted^24^.

#### Immunofluorescence and immunohistochemistry staining

Formalin-fixed, paraffin-embedded blocks of lung tissue from lung cancer patients or mouse tumor tissues were mounted on positively charged glass slides (R. Langenbrinck, Teningen, Germany). From each paraffin tissue block, 3 μm-thick sections were obtained. From each cryo tissue block, 5 μm-thick sections were obtained at −20 °C. Paraffin tumor tissue sections were rehydrated in three serial dilutions of xylene and decreasing concentrations of ethanol of 99.6%, 96% and 70%. Cryosections were permeabilized in ice cold acetone/methanol (1:1) at −20 °C for 30 min. After 10 min of rehydration in PBS at room temperature. After antigen retrieval, tissues were blocked with 5% BSA for 1 h at room temperature. The slides were then washed with PBS and incubated with the following primary antibodies overnight at 4 °C. Antibodies against the following markers were used: PCNA (1:100; Santa Cruz Biotechnology #sc7907), CD31 (1:100; BD Pharmingen #550274), F4/80 (1:100; eBioscience, BM8, #14-4801-82), Collagen I (1:70; Meridian Life Science® #T40777R), vWF (1:1200; Dako #A008229-2), CD206 (1:100; R&D Systems #AF2535-SP), TNF-α (1:100; Abcam #ab6671), ET1 (human 1:500, mouse 1:2000; abcam #ab117757), ETA (1:500; abcam #ab117521), and ETB (1:500; abcam #ab117529)Slides were rinsed three times with PBS and incubated with secondary antibody (Alexa488®, 1:1000, Invitrogen #A-11001 and Alexa555® 1:1000, Invitrogen #A-21428) for 1 h at room temperature. For double staining, incubation with an additional primary antibody was then carried out. For immunofluorescence, TOPRO-3-iodide (InvitrogenTM) was used for nuclear staining. Tissue slides were visualized with confocal microscopy (Zeiss LSM 710) with Zen 2011 software. For ET_1_, ET_A,_ and ET_B_ antibody binding was determined using alkaline phosphates (ZytoChem) with Tris-Wash buffer, TBS (ZytoChem). After extensive washing, sections were stained with permanent AP red kit (ZytoChem). The sections were counterstained with nuclear fast red and hematoxylin (Vector Laboratories).

#### PCNA positive cell quantification

Image processing and quantification was performed with HALO v3.3.2541 software. DAPI-based cell segmentation was used to improve phenotyping. The counts of cells with PCNA-positive in nuclei were normalized to the total cell counts.

#### Multiplexed immunofluorescence

All tumor sections (3 μm thick, as described above) were stained with Opal 7‐Color Automation IHC Kits (Akoya Bioscience) in the BOND‐RX Multiplex IHC Stainer (Leica). Each section was put through 3-6 sequential rounds of staining, which included blocking in 5% BSA followed by incubation with primary antibodies human; Pan-Cytokeratin, (1:200; abcam #ab7753, C-11); CD163 (1:250; abcam #ab182422, EPR19518), CD45 (1:200; abcam #ab10558), Collagen-1 (1:200; Abcam #ab34710), vWF (1:500; Dako #A008229-2), CCR2 (1:200; abcam #ab176390), CD162 (1:100; Biolegend #328802, KPL-1), CD44 (1:2000; Sigma #HPA005785), α-SMA (1:5000; Sigma #F3777, 1A4); mouse: CD206 (1:500; abcam; ab64693), F4/80 (1:500; Cell Signaling; 70076), TNF-α (1:200; abcam #ab6671), CCR2 (1:500; abcam #ab273050, EPR20844-15), CD44 (1:2000; abcam #ab157107), CD45 (1:500; Cell Signaling; 70257), CD163 (1:250; abcam #ab182422, EPR19518), Collagen-1 (1:500; Cell Signaling Technology #72026 S) corresponding secondary HRP-conjugated antibodies and Opal fluorophores as described^30,58^. Nuclei were counterstained with 4′,6‐diamidino‐2‐phenylindole (DAPI) and slides were mounted with Fluoromount‐G (SouthernBiotech). Imaging was performed with the VectraPolaris imaging system (Akoya Bioscience), and images were analyzed by using inForm software V2.4.10 (Akoya Bioscience). Distance measurements were made with HALO software v3.3.2541 (http://www.indicalab.com/halo#halo-modules).

#### Spheroid-based angiogenesis assay

Endothelial cell spheroids of defined cell number were generated^59^. In brief, HUVECs and HPMECs were suspended in culture medium containing 0.20% (w/v) methylcellulose (MO262; Sigma-Aldrich, Taufkirchen, Germany) and seeded by using the hanging drop method. Spheroids were formed within 24 h with a defined cell number (400 cells/ spheroid and were embedded into collagen (#08-115, Millipore, Temecula, CA, USA). The spheroid-containing gel was rapidly transferred into prewarmed 96 well plate (Sarstedt, Nümbrecht, Germany) and allowed to polymerize for 30 min prior adding conditioned media on top of the gel. After 24 h, pictures of the sprouting spheroids were taken with an inverted light microscope (Leica, Wetzlar, Germany). Sprouting was quantified by counting the number of the sprouts that had grown out of each spheroid using Fiji v1.5.2, with 5 spheroids analyzed per experimental group and experiment.

#### Endothelin ELISA

Concentrations of Endothelin in serum, cell culture supernatants and lysates were determined using an Endothelin-1 Quantikine Elisa Kit (R&D Systems, DET100), according to manufacturer’s instructions. Endothelin level were measured in duplicates using a microplate reader (Tecan, Männedorf, Switzerland).

#### Statistics and reproducibility

Statistical analyses were performed with the GraphPad Prism 9 Software (GraphPad Software, Inc.). One-way ANOVA followed by Fisher’s LSD post hoc test was used to compare the means of more than two independent groups; two independent groups were compared with the two-tailed student’s t-test with Welsh’s correction. Two-way ANOVA with Bonferroni’s multiple comparison test was used to evaluate the effect of two grouping variables e.g. tumor progression over time. Only *p*-values ≤ 0.05, considered as statistically significant were shown in graphs. Each experiment were independently repeated at least 3 times showing similar results.

### Reporting summary

Further information on research design is available in the Nature Research Reporting Summary linked to this article.

## Supplementary information

Supplementary Information

Reporting Summary

Supplementary Data 1

## Data Availability

The source data underlying all main and supplementary figures are provided as a source Data file. Bone marrow and lung tumor CD45^+^ cells scRNA-seq data generated in this study are publicly available and can be downloaded from the NCBI’s Gene Expression Omnibus under accession numbers. GSE206843 and GSE179191. Source data are provided with this paper.

## Source data

Source Data
