# Supplementary material for: Fibrocytes boost tumor-supportive phenotypic switches in the lung cancer niche via the endothelin system
Source: Nat Commun. 2022 Oct 14;13:6078. doi: 10.1038/s41467-022-33458-8 (PMC9568595; doi:10.1038/s41467-022-33458-8)
Supplement: Supplementary file 1 — Supplementary Information [file 41467_2022_33458_MOESM1_ESM.docx]

**Supplementary information**

Fibrocytes boost tumor-supportive phenotypic switches in the lung cancer niche via the endothelin system

Andreas Weigert^1,2†^, Xiang Zheng^3†^, Alina Nenzel^3†^, Kati Turkowski^3^, Stefan Günther^3^, Elisabeth Strack^1^, Evelyn Sirait-Fischer^1^, Eiman Elwakeel^1^, Ivan M. Kur^1^, Vandana S. Nikam^3^, Chanil Valasarajan^3^, Hauke Winter^4^, Alexander Wissgott^5^, Robert Voswinkel^3^, Friedrich Grimminger^5,6^, Bernhard Brüne^1,2^, Werner Seeger^3,5,6^, Soni Savai Pullamsetti^3,5,6^, Rajkumar Savai^2,3,5,6*^

^1^Goethe-University Frankfurt, Faculty of Medicine, Institute of Biochemistry I, Germany ^2^Frankfurt Cancer Institute (FCI), Goethe University, and German Cancer Consortium (DKTK), Partner Site Frankfurt, Frankfurt am Main, Germany

^3^Max Planck Institute for Heart and Lung Research, Member of the German Center for Lung Research (DZL), Member of the Cardio-Pulmonary Institute (CPI), Bad Nauheim, Germany

^4^Translational Lung Research Center (TLRC), Member of the DZL; Department of Thoracic Surgery, Thorax klinik at the University Hospital Heidelberg, Heidelberg, Germany

^5^Department of Internal Medicine, Justus-Liebig University Giessen, Member of the DZL, Member of CPI, Giessen, Germany

^6^Institute for Lung Health (ILH), Justus Liebig University, Giessen, Germany

*Corresponding author: [rajkumar.savai@mpi-bn.mpg.de](mailto:rajkumar.savai@mpi-bn.mpg.de)

^†^These authors contributed equally to this work.

**Supplementary Figure 1. Collagen, fibrocytes, and monocyte-macrophage genes in CD45^+^ cells scRNAseq and fibrocyte depletion does not affect other BM cells. (A)** Collagen genes, **(B)** fibrocytes and monocyte-macrophage genes are summarized by median or mean expression per group by dot plot respectively. **(C)** Schematic diagram of HSV-TK/Col1 transgene generation. HSV-TK: herpes simplex virus thymidine kinase, IRES: internal ribosome entry site, GFP: green fluorescent protein. **(D)** Quantification of CD45^–^ cells, CD45^+^ cells, lymphocytes (CD45^+^CD11b^–^), B cells (CD45^+^CD11b^–^B220^+^), neutrophils (CD45^+^CD11b^+^Ly6G^+^) and macrophages (CD45^+^CD11b^+^Ly6G^–^F4/80^+^) in fibrocyte-depleted mice (HSV-TK/Col1+Ganciclovir) and control mice (HSV-TK/Col1). Statistical test was performed using two-tailed unpaired t–test with Welch’s correction, n=4. Source data are provided in the source data file.

**Supplementary Figure 2. Fibrocyte depletion reduces tumor growth and alters tumor microenvironment.** **(A)** Fibrocyte-depleted mice (HSV-TK/Col1+Ganciclovir) or control mice (HSV-TK/Col1+PBS) were subcutaneously (s.c.) injected with LLC1 cells. **(B)** Tumor size (left) and representative photographic images (right; scale bar, 1mm). *P* values were determined using two-way ANOVA with Fisher’s LSD test, n=8. **(C)** Tumor weight on day 20 in the s.c. tumor model using fibrocyte-depleted and control mice. *P* values were determined using two-tailed unpaired t-test with Welch’s correction, n=8. **(D–F)** Analysis of tumor tissue from tumor bearing mice as in **C**. **(D)** Representative immunofluorescence photomicrographs of PCNA stained (proliferation marker, red) and TOPRO-stained (nuclei, blue), n=10. scale bar, 25 µm. **(E)** Representative immunofluorescence images of vascular marker (CD31, green) and TOPRO-stained nuclei (blue), n=10. scale bar, 50 µm. **(F)** Representative immunofluorescence photomicrographs of macrophage marker (F4/80, green) and TOPRO-stained nuclei (blue), n=10. scale bar, 25 µm. **(G)** Representative composite images of lung tumor tissues with multiplex immunofluorescence staining. Individual markers in composite images are Col1 (green), CCR2 (orange), CD44 (blue), CD163 (yellow), CD45 (red) and DAPI (white). Cell types marked by arrows, n=10. scale bar, 50 μm. **(H)** Representative composite images of lung tumor tissues with multiplex immunofluorescence staining. Individual markers in the framed area of composite images are F4/80 (green), CD206 (yellow), TNF-α (red) and DAPI (white). Cell types marked by arrows, n=10. scale bar, 50 μm. Source data are provided in the source data file.

**Supplementary Figure 3.** **Fibrocyte depletion reduces lung tumor progression in a KRas^LA2^ oncogenic mouse model.** **(A)** Representative photographs of whole lungs (upper panel, scale bar, 2 mm) and H&E staining (down panel) of lung sections (scale bar, 1.25 mm) from KRas^LA2^ oncogenic model using fibrocyte-depleted (HSV-TK/Col1+Ganciclovir), control mice (HSV-TK/Col1 and control), n=4. **(B)** Quantification of macroscopic and microscopic lung tumor nodules in the KRas^LA2^ lung tumor model from fibrocyte-depleted and control mice lung tumors, n=4. **(C)** Quantification of PCNA^+^ proliferating cells (n=4, 5 images per lung tumor) and vWF^+^ vessels (n=4, 5 images per lung tumor) in the KRas^LA2^ lung tumor model from fibrocyte-depleted, and control mice lung tumors by immunohistochemistry. **(D)** Quantification of fibrocytes (CD45^+^CD162^+^F4/80^+^CD9^+^CCR2/5^+^) and macrophages (CD45^+^CD162^+^F4/80^+^CD9^–^CCR2/5^–^) in KRas^LA2^ lung tumor model from fibrocyte-depleted, and control mice lung tumors by FACS analysis, n=3. **(E)** Quantification of fibroblasts (CD45^-^CD163^–^Col1^+^), fibrocytes (CD45^+^CD163^+^CCR2^+^Col1^+^), and macrophages (CD45^+^CD163^+^CCR2^–^Col1^–^), M2-like (CD206^+^) and M1-like (TNF^+^) macrophages in the KRas^LA2^ lung tumor model from fibrocyte-depleted (HSV-TK/Col1+Ganciclovir), HSV-TK/Col1 and control mice lung tumors by multiplex immunofluorescence, n=3. (**B, C, D, E**) *P* values were determined using one-way ANOVA with Fisher’s LSD test. Source data are provided in the source data file.

**Supplementary Figure 4.** **Human PBMC-derived fibrocyte characterization. (A)** Human fibrocytes were isolated from PBMCs*.* Representative flow cytometry plots show fibrocyte gating using CD45^+^, CCR2^+^, CD33^+^, CXCR3^+^ and CCR5^+^. **(B)** Representative FACS histograms show expression of Col1, CD44 and CD162 on fibrocytes (CD45^+^CD33^+^CXCR3^+^CCR2^+^CCR5^+^), CCR5^–^ monocytes (CD45^+^CD33^+^CCR2^+^CCR5^–^CXCR3^–^), CCR5^+^ monocytes (CD45^+^CD33^+^CCR2^+^CCR5^+^CXCR3^–^) and lymphocytes (CD45^+^CD33^–^).

**Supplementary Figure 5. Co-injection of fibrocytes and fibroblasts with human H226 and H1650 lung cancer cells increases tumor growth.** Co-injection of human lung cancer H226 and H1650 cells with fibrocytes and fibroblasts into BALB/c nude mice. **(A)** Tumor growth curve (left panel) over 16 days of H226 or H1650 and H226+fibroblast or H226+fibrocytes and H1650+fibroblast or H1650+fibrocytes, n=5. Representative tumor pictures (right panel) at day 16 (scale bar 2 mm). **(B)** Tumor weight of tumors derived from injected H226 and H1650 cancer cells alone or H226+fibroblast and H1650+fibroblast or H226+fibrocytes and H1650+fibrocytes after 16 days, n=5. **(C, D)** Quantification of PCNA^+^ proliferating cells (n = 5, 5 images per tumor) and vWF^+^ vessels (n= 5, 5 images per tumor) in tumor tissues from injected H226 and H1650 cancer cells alone or H226+fibroblast and H1650+fibroblast or H226+fibrocytes and H1650+fibrocytes by immunohistochemistry. **(E, F)** Quantification of fibrocytes (CD45^+^CD11b^+^CD162^+^F4/80^+^ CCR2/5^+^CD9^+^CD44 Col1^+^) and macrophages (CD45^+^CD11b^+^CD162^+^F4/80^+^CCR2/5lo/^–^CD9^–^) in tumors derived from injected H226 and H1650 cancer cells alone or H226+fibroblast and H1650+fibroblast or H226+fibrocytes and H1650+fibrocytes by FACS analysis, n=5. Quantification of **(G)** fibroblasts (CD45^–^CD163^–^Col1^+^), **(H)** fibrocytes (CD45^+^CD163^+^CCR2^+^Col1^+^), **(I)** macrophages (CD45^+^CD163^+^CCR2^–^Col1^–^), **(J)** M2-like (CD206^+^) and **(K)** M1-like (TNF^+^) in tumors derived from injected H226 and H1650 cancer cells alone or H226+fibroblast and H1650+fibroblast or H226+fibrocytes and H1650+fibrocytes by multiplex immunofluorescence, n=3. **(A)** *p* values were determined using two-way ANOVA with Bonferroni’s multiple comparison. **(B-K)** *p* values were determined using one-way ANOVA with Fisher’s LSD test. Source data are provided in the source data file.

**Supplementary Figure 6. Cross-talk between fibrocytes and cancer cells increases cancer cell proliferation, migration and influences the fibrocyte phenotype.** Different lung cancer cell lines (H226, H1650, A427 or HCC15) were incubated with conditioned medium (CM) from each cancer cell line alone, fibrocytes alone and Fibrocytes+cancer cells (H226, H1650, A427 or HCC15) and were assessed for **(A)** proliferation (n=3 independent experiments, 8 technical replicates) and **(B)** migration (n=3 independent experiments, 3 technical replicates). **(C-F)** mRNA expression of *Col1A1, Col3A1* and *FN1* in fibrocytes alone and in fibrocytes co-cultured with cancer cells **(C)** H226, **(D)** H1650, **(E)** A427 and **(F)** HCC15, n=6. (**A, B**) *p* values were determined using one-way ANOVA with Fisher’s LSD test and (**C-F**) *p* values were determined using two-tailed unpaired t-test with Welch’s correction. Source data are provided in the source data file.

**Supplementary Figure 7. Cross-talk between fibrocytes and cancer cells increases VEGF expression. (A)** *VEGF* mRNA expression in cancer cells alone or in cancer cells (H226, H1650, A427 and HCC15)+fibrocyte co-cultures, n=6. *P* values were determined using two-tailed unpaired t-test with Welch’s correction. Source data are provided in the source data file.

**Supplementary Figure 8. Cytokine profile in cancer and fibrocytes cocultures.** **(A)** Visualization of antibody spotted membranes after incubation with different CM from co-cultured with A549 and fibrocytes. **(B)** Secretory factors of cancer cells and fibrocytes and co-cultured CM. **(C)** Quantification of cytokines (IL-23, IFNγ, CD54, IL-1ra, IL-6, CCL5, CXCL10, MIF, IL-8, CXCL1, Serpin E1, CCL2) secreted in fibrocytes, cancer cells and co-cultures.

**Supplementary Figure 9.** **Cross-talk between fibrocytes and cancer cells influences the endothelin pathway.** **(A)** mRNA expression of *ET_1_, ET_A_* and *ET_B_* in fibrocytes alone and fibrocytes co-cultured with cancer cells (H1650, A427, HCC15, H226), n=6. **(B)** mRNA expression of *ET_1_, ET_A_* and *ET_B_* in cancer cells alone and cancer cells (A549, H226, H1650, A427, HCC15) co-cultured with fibrocytes, n=6. **(C)** ET1 levels of fibrocytes, cancer cells, endothelial cells (HUVECs) alone and fibrocytes+cancer cells or fibrocytes+HUVEC or Fibrocytes+HUVEC+cancer cells co-cultures, n=3. (**A, B**) *p* values were determined using two-tailed unpaired t-test with Welch’s correction and (**C**) *p* values were determined using one-way ANOVA with Fisher’s LSD test. Source data are provided in the source data file.

Supplementary Figure 10. ET_A_ and ET_B_ expression in co-cultured cells. Representative flow cytometry plots show the ET_A_ and ET_B_ expression in cancer cells (A549), FMO (fluorescence minus one), fibrocytes, macrophages alone or co-cultures of cancer cells and fibrocytes, and triple co-cultures of cancer cells, fibrocytes and macrophages. Cell counts are shown.

**Supplementary Figure 11.** **Cross-talk between fibrocytes and cancer cells has no effect on PDGF, IGF and EGF pathways and is not dependent on TGFβ.** mRNA expression of **(A)** *PDGFA, PDGFB* and *PDGFC*, **(B)** *IGF2* and *IGF1R*, **(C)** *EGF* and *EGFR,* **(D)** and *TGFß1* in A549 or A549+fibrocytes, n=4. **(E)** *ET_1_, ET_A_* and *ET_B_* mRNA expression in A549 cells alone or A549+fibrocytes in presence or absence of SB-525334, n=3. **(F)** ET1 measurement in CM of A549 or A549+fibrocytes in presence or absence of SB-525334, n=3. (**A-D**) *p* values were determined using two-tailed unpaired t-test with Welch’s correction and (**E, F**) *p* values were determined using one-way ANOVA with Fisher’s LSD test. Source data are provided in the source data file.

**Supplementary Figure 12.** **ET_1_, ET_A_ and ET_B_ are upregulated in human and mouse lung cancer tissues.** **(A)** Representative immunoreactivity photomicrographs of ET_1_, ET_A_ and ET_B_ in lung tissues from healthy human donors and in lung cancer tissues (upper: lung adenocarcinoma (ADC); lower: lung squamous carcinoma (SSC), n=3, scale bar, 20 µm. **(B)** Representative immunoreactivity photomicrographs of ET_1_, ET_A_ and ET_B_ in mouse normal lung and lung cancer tissues (upper: KRas^LA2^ lung tumor; lower: LLC1 lung tumor), n=3, scale bar, 20 µm. Endothelin (ET_1_) levels were measured in plasma from **(C)** healthy and lung cancer patients, n=8. **(D)** Endothelin levels of fibrocyte-depleted (HSV-TK/Col1+Ganciclovir), control mice (HSV-TK/Col1 and control) plasma samples were measured by ELISA, n=3. **(E)** Endothelin levels from mice injected with A549 or co-injected with fibrocytes and fibroblasts plasma samples were measured by ELISA, n=3. (**C**) *p* values were determined using two-tailed unpaired t-test with Welch’s correction and (**D, E**) *p* values were determined using one-way ANOVA with Fisher’s LSD test. Source data are provided in the source data file.

**Supplementary Figure 13. IGFBP-2, IGFBP-3, VEGF, PDGF-AA, IL6, IL31 impact the endothelin system in cancer cells. (A)** A cytokine array consisting of 105 cytokines was used to examine the secretome profile of A549 cell and fibrocyte co-cultures in the presence and absence of bosentan treatment. Heatmaps show the top down regulated (upper heatmap) up regulated (down heatmap) cytokines after bosentan treatment. **(B)** *ET_1_, ET_A_* and *ET_B_* mRNA expression in cancer cells (A549) after IGFBP-2, IGFBP-3, VEGF, PDGF-AA, IL6, IL31 cytokine stimulation. n=6. **(C)** *ET_1_, ET_A_* and *ET_B_* mRNA expression in cancer cells (A549) after addition of A549-fibrocyte co-culture CM with/without neutralizing antibodies (IGFBP-2, IGFBP-3, VEGF, PDGF-AA, IL6, IL31), n=6. (**B, C**) *p* values were determined using one-way ANOVA with Fisher’s LSD test. Source data are provided in the source data file.

**Supplementary Figure 14.** **Bosentan treatment reduces tumor growth in mouse tumor model.** Placebo (DMSO) or bosentan was administered to LLC1-injected C57BL/6 mice for 16 days. **(A)** Tumor growth curve over 16-days period of LLC1 (placebo) and LLC1+bosentan s.c. tumors, n=5. Representative images of s.c. tumors at end of the experiment at day 16, scale bar, 2 mm. **(B)** Tumor weight of LLC1 and LLC1+bosentan s.c. tumors measured on day 16, n=5. **(A)** *p* values were determined using two-way ANOVA with Bonferroni’s multiple comparison. **(B)** *p* values were determined using two-tailed unpaired t-test with Welsh’s correction. Source data are provided in the source data file.
