# Supplementary material for: Fibrocytes boost tumor-supportive phenotypic switches in the lung cancer niche via the endothelin system
Source: Nat Commun. 2022 Oct 14;13:6078. doi: 10.1038/s41467-022-33458-8 (PMC9568595; doi:10.1038/s41467-022-33458-8)
Supplement: Supplementary file 2 — Reporting Summary [file 41467_2022_33458_MOESM2_ESM.pdf]

nature research

Corresponding author(s): Rajkumar Savai

Last updated by author(s): Aug 29, 2022

Reporting Summary

Nature Research wishes to improve the reproducibility of the work that we publish. This form provides structure for consistency and transparency in reporting. For further information on Nature Research policies, see our Editorial Policies and the Editorial Policy Checklist.

Statistics

For all statistical analyses, confirm that the following items are present in the figure legend, table legend, main text, or Methods section.

n/a	Confirmed	
[ ]	[x]	The exact sample size (n) for each experimental group/condition, given as a discrete number and unit of measurement
[ ]	[x]	A statement on whether measurements were taken from distinct samples or whether the same sample was measured repeatedly
[ ]	[x]	The statistical test(s) used AND whether they are one- or two-sided<br>*Only common tests should be described solely by name; describe more complex techniques in the Methods section.*
[x]	[ ]	A description of all covariates tested
[ ]	[x]	A description of any assumptions or corrections, such as tests of normality and adjustment for multiple comparisons
[ ]	[x]	A full description of the statistical parameters including central tendency (e.g. means) or other basic estimates (e.g. regression coefficient) AND variation (e.g. standard deviation) or associated estimates of uncertainty (e.g. confidence intervals)
[ ]	[x]	For null hypothesis testing, the test statistic (e.g. F, t, r) with confidence intervals, effect sizes, degrees of freedom and P value noted<br>*Give P values as exact values whenever suitable.*
[x]	[ ]	For Bayesian analysis, information on the choice of priors and Markov chain Monte Carlo settings
[x]	[ ]	For hierarchical and complex designs, identification of the appropriate level for tests and full reporting of outcomes
[x]	[ ]	Estimates of effect sizes (e.g. Cohen's d, Pearson's r), indicating how they were calculated

Our web collection on statistics for biologists contains articles on many of the points above.

Software and code

Policy information about availability of computer code

Data collection	StepOne™ and StepOnePlu ™Software v2.3 for qRT-PCR. Nextseq500 (illumina)for Single Cell Sequencing. BOND-RX Multiplex IHC Stainer (Leica) for Multiplexed immunofluorescence, Imaging was performed with the VectraPolaris imaging system (Akoya Bioscience), and images were analyzed by using inForm software V2.4.10 (Akoya Bioscience). Image processing quantification and distance measurements were performed with HALO software (http://www.indicalab.com/halo#halo-modules). Immunofluorescence were visualized with confocal microscopy (Zeiss LSM 710) with Zen 2011 software. Image J (Fiji) Software with Angiogenesis Analyzer plugin was used for tube formation anlysis. Infinite M 200 Pro from Tecan for BrdU and ELISA measurements. FACSAria III Cell Sorter (BD) was used for cell separation. LSRII/ Fortessa flow cytometer (BD) used for Protein expression analysis. For Cytokine Array, Amersham Hyperfilm ECL films (GE Healthcare Europe GmbH) was used and analysis and quantification were performed with the BioDoc Analyze Software v2.67.5.0 (Biometra). Nanozoomer 2.0HT digital slide scanner C9600 (Hamamatsu Photonics) was used for migration analysis. Inverted light microscope (Leica) was used for visualization of sprouts.
Data analysis	STARsolo v2.7.3a, Scanpy v1.6 and cellxgene v1.0.0 were used for ScRNA-seq data analysis. inForm software V2.4.10 (Akoya Bioscience) and HALO software 3.3.2541 (http://www.indicalab.com/halo#halo-modules) were used for image analysis. Excel (Microsoft) and Prism 9 (GraphPad inc) was used for graph generation and data analysis. Flow cytometry data were acquired using FACSDiva software v9.0 (BD Biosciences) and analyzed using FlowJo software V10 (Treestar). ImageJ (Fiji) v1.5.2 was used to quantify tube formation, migration and sprouting.

For manuscripts utilizing custom algorithms or software that are central to the research but not yet described in published literature, software must be made available to editors and reviewers. We strongly encourage code deposition in a community repository (e.g. GitHub). See the Nature Research guidelines for submitting code & software for further information.

Data

Policy information about availability of data

All manuscripts must include a data availability statement. This statement should provide the following information, where applicable:

- Accession codes, unique identifiers, or web links for publicly available datasets
- A list of figures that have associated raw data
- A description of any restrictions on data availability

ScRNA-seq data have been deposited in Gene Expression Omnibus number GEO: GSE179191;https://www.ncbi.nlm.nih.gov/geo/query/acc.cgi?acc=GSE179191 and GSE206843; https://www.ncbi.nlm.nih.gov/geo/query/acc.cgi?acc=GSE206843.

Field-specific reporting

Please select the one below that is the best fit for your research. If you are not sure, read the appropriate sections before making your selection.

☒ Life sciences ☐ Behavioural & social sciences ☐ Ecological, evolutionary & environmental sciences

For a reference copy of the document with all sections, see nature.com/documents/nr-reporting-summary-flat.pdf

Life sciences study design

All studies must disclose on these points even when the disclosure is negative.

Sample size	Sample size was estimated based on previous experience with the experimental approaches. For all tumor models at least 4-5 mice are required for growth analysis and tumor weight/burden. For Proliferation and vessel counts at least 4 mice are required. For FACS analysis at least 3 mice are required. Sample size were determined based on established practice and applicable standards. Each experiment in which data were quantified was performed with at least 3 replicates.
Data exclusions	No data were excluded from the analysis.
Replication	All experiments were replicated independently for at least three times to ensure reproducibility. . All in vivo studies were performed with indicated number of animals. Sample sizes and statistical analyses and significance levels are all indicated in the figure legends or the method part.
Randomization	Mice were allocated into experimental groups based on genotype or treatment.
Blinding	Numeric values were assigned to mice and human sample. Researchers were blinded to the experimental groups of the immunofluorescence histology samples during the counting of vessels and Ki67+ cells. Otherwise, researchers were not blinded because it was necessary to allocate samples to experimental groups based on differences intrinsic to the samples. The data were analyzed by at least two different individuals.

Reporting for specific materials, systems and methods

We require information from authors about some types of materials, experimental systems and methods used in many studies. Here, indicate whether each material, system or method listed is relevant to your study. If you are not sure if a list item applies to your research, read the appropriate section before selecting a response.

Materials & experimental systems

n/a	Involved in the study	
☐	☒	Antibodies
☐	☒	Eukaryotic cell lines
☒	☐	Palaeontology and archaeology
☐	☒	Animals and other organisms
☒	☐	Human research participants
☒	☐	Clinical data
☒	☐	Dual use research of concern

Methods

n/a	Involved in the study	
☒	☐	ChIP-seq
☐	☒	Flow cytometry
☒	☐	MRI-based neuroimaging

Antibodies

Antibodies used	Antibodies for staining murine bone marrow and tumor samples were: B220-APC/Fire 750 (Biolegend #103260, RA3-6B2: 1:100), CCR2-PE-Cy7 (Biolegend #150612, SA203G11, 1:100), CCR5-PE-Cy7 (Biolegend #107018, HM-CCR5, 1:50), CXCR3-BV711 (BD Biosciences #740825, CXCR3-173, 1:100), CD9-BV650 (BD Biosciences #564236, KMC8, 1:100), CD11b-BV605 (BD Biosciences #563015, M1/70, 1:200), CD44-AlexaFluor 700 (BD Biosciences # 560567, IM7, 1:100), CD45-Vioblue (Miltenyi Biotec #130-118-953, 30F11, 1:50), CD162-BV510 (BD Biosciences #563448, 2PH1, 1:200), CD326/Epcam-BV711 (BD Biosciences #563134, G8.8, 1:100),

F4/80-PE-Cy7 (Biolegend # 123114, BM8, 1:100), Ly-6C-PerCP-Cy5.5 (Biolegend, AL-21, 1:200), Ly-6G-APC-Cy7 (Biolegend #127624, 1A8, 1:100), RANKL-AlexaFluor 647 (BD Biosciences #560296, IK22-5, 1:50), Sca-1-PE-CF594 (BD Biosciences # 562730, D7, 1:100), Siglec H-APC (Biolegend # 129612, 551, 1:50). Antibodies used for staining human PBMCs, fibrocytes, fibroblasts and macrophages were CCR2-PE-Cy7 (Biolegend #357212, K036C2,1:50), CCR5-PE (Biolegend #359106, J418F1, 1:50), CXCR3-BV711 (BD Biosciences #563156, 1C6, 1:100), CD14-APC H7 (BD Biosciences #560180, Møp9), CD33-BV510 (BD Biosciences #563257, WM53), CD44-APC (Biolegend #338806, BJ18,1:200), CD45-AlexaFluor 700 (BD Biosciences #560566, HI30, 1:50), CD64-BV605 (BD Biosciences #740406, 10.1, 1:100), CD162-APC/Fire 750 (Biolegend #328814, KPL-1, 1:100), CD163-BV421 (BD Biosciences #566277, GHI/61, 1:100), CD163-FITC (Biolegend #333618, GHI/61, 1:100), HLA-DR-APC (Biolegend #980406, L243, 1:200) and MERTK-BV421 (Biolegend #367604, 590H11G1E3, 1:50).

Expression of endothelin receptors on cells from co-cultures was determined using EDNRA-AlexaFluor 405 (R&D Systems #FAB65381V-100UG, 485709, 1:50) and EDNRB-AlexaFluor 750 (R&D Systems #FAB4496S-100UG, 671917, 1:50) antibodies. Intracellular human and mouse collagen I was stained with polyclonal Collagen-I-FITC antibodies (ThermoFisher Scientific, #600-402-103, 1:100), anti-human S100A8 with S100A8-DyLight 594 antibody (Novus Biologicals #NBP2-27067DL594, 63N13G5, 1:200).

Neutralizing antibodies: IL-31 (2 µg/mL R&D Systems #AF2824), IL-6 (1 µg/mL R&D Systems #MAB2061-100), PDGF-AA (2 µg/mL R&D Systems #AB-221-NA), VEGF (0.1 µg/mL R&D Systems #MAB293-100), IGFBP-2 (2 µg/mL R&D Systems #AF674), and IGFBP-3 (1 µg/mL R&D Systems #AF675)

Antibodies for IF and IHC: PCNA (1:100; Santa Cruz Biotechnology #sc7907), CD31 (1:100; BD Pharmingen #550274), F4/80 (1:100; eBioscience, BM8, #14-4801-82), Collagen I (1:70; Meridian Life Science® #T40777R), vWF (1:1200; Dako #A008229-2), CD206 (1:100; R&D Systems #AF2535-SP), TNF-α (1:100; Abcam #ab6671), ET1 (human 1:500, mouse 1:2000; abcam #ab117757), ETA (1:500; abcam #ab117521), and ETB (1:500; abcam #ab117529) and secondary antibody (Alexa488®, 1:1000, Invitrogen #A-11001 and Alexa555® 1:1000, Invitrogen #A-21428).

Multiplexed immunofluorescence used antibodies: human; Pan-Cytokeratin, (1:200; abcam # ab7753, C-11); CD163 (1:250; abcam #ab182422, EPR19518), CD45 (1:200; abcam #ab10558), Collagen-1 (1:200; Abcam #ab34710), vWF (1:500; Dako #A008229-2), CCR2 (1:200; abcam #ab176390), CD162 (1:100; Biolegend #328802, KPL-1), CD44 (1:2000; Sigma #HPA005785), α-SMA (1:5000; Sigma #F3777, 1A4); mouse: CD206 (1:500; abcam; ab64693), F4/80 (1:500; Cell Signaling; 70076), TNF-α (1:200; abcam #ab6671), CCR2 (1:500; abcam #ab273050, EPR20844-15), CD44 (1:2000; abcam #ab157107), CD45 (1:500; Cell Signaling; 70257), CD163 (1:250; abcam #ab182422, EPR19518), Collagen-1 (1:500; Cell Signaling Technology #72026S), corresponding secondary HRP-conjugated antibodies and Opal fluorophores.

Antibodies used for fibrocyte generation: CD2 (Merck #MABF83, clone RPA-2.10) 1 µg/µl; CD14 (Merck #MAB1219, clone 2D-15C) 0,5 µg/µl; CD19 (Merck #MAB1794, clone FMC63) 0,1 µg/µl.

Validation

FMO controls were used for antibody validation in flow cyometry. IHC antibodies were routinely used. All monoclonal antibodies used are commercially available and were validated with mouse cells for flow cytometry application by the manufacturers. A validation statement for each antibody is available on the manufacturer's website. BD Bioscience (https://www.bdbiosciences.com)
BioLegend (https://www.biolegend.com)
Miltenyi Biotec (https://www.miltenyibiotec.com/AU-en/products/macs-antibodies/antibodies-for-flow-cytometry.html)
Abcam (https://www.abcam.com)

Eukaryotic cell lines

Policy information about cell lines

Cell line source(s)

A549 (CCL-185), A427 (HTB-53), H1650 (CRL-5883) , H226 (CRL-5826) and LLC1 (CRL-1642) were purchased from ATCC. HCC15 (ACC 496) cells were purchased from DSMZ. HUVECs (PB-CH-190-8013) were obtained from PELOBiotech GmbH, HPMECs (C-12281) were obtained from PromoCell.

Authentication

None of these cell lines used were authenticated.

Mycoplasma contamination

All cell lines tested negative for mycoplasma contamination.

Commonly misidentified lines (See ICLAC register)

No commonly misidentified lines were used.

Animals and other organisms

Policy information about studies involving animals; ARRIVE guidelines recommended for reporting animal research

Laboratory animals

C57BL/6 mice, HSV-TK/Col1 and immunodeficient BALB/c nude mice (CAnN.Cg-Foxn1nu/Crl) were purchased from Charles River (Sulzfeld, Germany) (male and female, 16 weeks old) and transgenic line KRasLA2 was purchased from Jackson Laboratory (Bar Harbor, ME, USA) (females and males, 12 weeks old). The transgenic lines HSV-TK/Col1+KRasLA2 and C57BL/6/KRasLA2 mice were generated in our laboratory (females and males, 12 weeks old).
Male and female, were bred (intercrossed in some cases) and housed in Specific-Pathogen-Free environments on a 12 hour light-dark cycle at 30-70 % humidity and temperature of 20-26°C, with ad libitum access to water and standard chow.

Wild animals

The study did not involve wild animals.

Field-collected samples

The study did not involve samples collected from the field.

Ethics oversight

Local authorities (Regierungspräsidium Darmstadt, Hessen, Germany) approved all animal studies. All mice were kept under specific-

pathogen-free conditions and handled in accordance with the European Union Commission on Laboratory Animals.

Note that full information on the approval of the study protocol must also be provided in the manuscript.

Flow Cytometry

Plots

Confirm that:

- [x] The axis labels state the marker and fluorochrome used (e.g. CD4-FITC).
- [x] The axis scales are clearly visible. Include numbers along axes only for bottom left plot of group (a 'group' is an analysis of identical markers).
- [x] All plots are contour plots with outliers or pseudocolor plots.
- [x] A numerical value for number of cells or percentage (with statistics) is provided.

Methodology

Sample preparation	Single-cell suspensions from tumor tissue samples were obtained by using the mouse Tumor Dissociation kit (Miltenyi Biotec, CA, USA). PBMCs from healthy patients and from patients with lung cancer were isolated by density-gradient centrifugation over Ficoll-Paque and were then used to assess the number of circulating fibrocytes.
Instrument	Samples were analyzed using an LSRII Fortessa cell analyzer or sorted using a FACSymphony S6 cell sorter (both from BD Biosciences)
Software	Data were collected using FACSDiva software (BD Biosciences) and analyzed using FlowJo software V10 (Treestar).
Cell population abundance	The abundance of cell populations in post-sort fractions was > 95%, which was controlled by post-sort analysis.
Gating strategy	For analysis of bone marrow cells, FSC/SSC gating was performed focussing on monocytic/lymphocyte/progenitor fraction, followed by doublet discrimination. After gating for CD45+ cells, fibrocytes were agted as SiglecH–, CD162+, CD9+, CD11b+, CD44+, CCR2/5+ cells, monocytes were gated as CD11b+ Ly6G- Ly6C+ cells, lymphocytes as CD11b– cells, B cells as CD11b– B220+, neutrophils as CD11b+ Ly6G+ and macrophages as CD11b+ Ly6G– F4/80+ cells.<br>Cell subsets in tumors were pregated by focussing on living sinlge cells. Fibrocytes were identified as CD45+ CD11b+ CD162+ F4/80+ CCR2/5+ CD9+ CD44+ Col1+, monocytes as CD45+ CD11b+ Ly6C+, macrophages as CD45+ CD11b+ CD162+ F4/80+ CCR2/5lo/– CD9–, neutrophils as CD45+ CD11b+ Ly6G+, and osteoblast-like cells as CD45– CD44+ Sca1+ CD9+ RANKL+.<br>Human fibrocytes were gated from PBMCs focussing on CD45+ SSC low cells. Fibrocytes were either defined as CD45+ Col1+ or more specifically as CD45+, CCR2+, CD33+, CXCR3+, CCR5+ Col1+ CD162+ CD44+ cells. Monocytes were CD45+, CD33+, CXCR3+, CCR2+ CCR5+/-, lymphocytes were CD45+ CD33–.<br>FMO controls were used for gating.

- [x] Tick this box to confirm that a figure exemplifying the gating strategy is provided in the Supplementary Information.
